# Environment-Induced Reversible Modulation of Optical and Electronic Properties of Lead Halide Perovskites and Possible Applications to Sensor Development: A Review

**DOI:** 10.3390/molecules26030705

**Published:** 2021-01-29

**Authors:** Maria Luisa De Giorgi, Stefania Milanese, Argyro Klini, Marco Anni

**Affiliations:** 1 Dipartimento di Matematica e Fisica “Ennio De Giorgi”, Università del Salento, Via per Arnesano, 73100 Lecce, Italy; stefania.milanese@studenti.unisalento.it (S.M.); marco.anni@unisalento.it (M.A.); 2 Institute of Electronic Structure and Laser, Foundation for Research and Technology-Hellas, P.O. Box 1385, Heraklion, 71110 Crete, Greece; klini@iesl.forth.gr

**Keywords:** lead halide perovskites, sensors, photoluminescence, amplified spontaneous emission, thin films

## Abstract

Lead halide perovskites are currently widely investigated as active materials in photonic and optoelectronic devices. While the lack of long term stability actually limits their application to commercial devices, several experiments demonstrated that beyond the irreversible variation of the material properties due to degradation, several possibilities exist to reversibly modulate the perovskite characteristics by acting on the environmental conditions. These results clear the way to possible applications of lead halide perovskites to resistive and optical sensors. In this review we will describe the current state of the art of the comprehension of the environmental effects on the optical and electronic properties of lead halide perovskites, and of the exploitation of these results for the development of perovskite-based sensors.

## 1. Introduction

The field of the development of chemical sensors has undergone a significant expansion in the last decade and it is currently one of the most active research area in nanoscience. The possible applications of gas sensors are extremely broad, and include, for example, the detection of air pollutants for environmental monitoring, of explosive vapors for security control and of toxic gases hazardous to human health [1,2,3,4,5].

Among the possible active materials, thin films of semiconductors are particularly promising, due to the possibility to tune electrical and/or optical properties when interacting with the gas analyte. The main properties of ideal sensors are the sensitivity, the selectivity and the reversibility which permit one to detect very low concentrations of gases (target gases), discriminate among them and come back to the pristine stage when the exposition is over. Another crucial issue is a fast response to the stimulus induced by the interaction of the gas with the sensing element and a high recovery speed. The possibility to use the sensor in simple working conditions, moreover, requires the capability to operate at room temperatures without the need for an external trigger, and good stability to external conditions; low-cost and facile fabrication processes are also important for the development of cheap devices with a long operational lifetime.

To date, sensing has been achieved almost entirely by examining changes in the electrical conductance of properly prepared nanostructures of semiconducting materials exposed to different environments [6]. The typical active materials are metal oxides, such as ${\mathrm{SnO}}_{2},\mathrm{ZnO},{\mathrm{TiO}}_{2},{\mathrm{Co}}_{3}O_{4}$ and FeO, which need elevated temperatures (200–400 ${}^{{}^{\circ}}C)$ to operate [7]. This feature limits the application of these materials for the detection in flammable or explosive atmospheres and the investigation of biomaterials. A further drawback of metal oxide resistive sensors is the typical need for pre-treatments in order to induce the sensing properties and the need for expensive deposition techniques for the realization of the sensing devices [8].

For these reasons, the research aiming to develop novel active materials for sensing, able to combine cheap deposition, high sensitivity and the capability to operate at room temperature is still running.

Among the different classes of innovative semiconducting materials, lead halide perovskites recently started to receive enormous interest thanks to their unique capability to combine simple film deposition from a solution, active properties typical of semiconductors and wide chemical flexibility [9,10,11,12]. The general chemical formula of lead halide perovskites is APbX${}_{3}$, where X = Cl, Br or I, or mixed Cl/Br and Br/I systems, and A is an organic group in hybrid organic–inorganic perovskites or an inorganic cation in totally inorganic ones.

The chemical and physical properties of the perovskites strongly depend on the preparation procedures. APbX${}_{3}$ are typically obtained by two methods: synthesis from solution and deposition from vapor phase. Alternative procedures, even if less common, are through solid-state reactions [13] or atomic-layer-deposition (ALD) [14]. In the precipitation-from-solution procedure, the precursors (PbX${}_{2}$ and AX) are simultaneously dissolved in a solution; the subsequent evaporation of the solvent, by heating or spin-coating, leads to the formation of the perovskite crystals (one step method). In the two-step procedure, instead, a solution containing the lead halide salt is spin-coated onto a substrate and dried in order to obtain a PbX${}_{2}$ film. Then the film is exposed to AX (e.g., dipped in a AX-based solution) to convert the PbX${}_{2}$ film into the perovskite. In the vapor-phase deposition the perovskite is obtained by the coevaporation of the lead halide salt and the AX halide. This method generally yields more uniform films if compared with those synthesized from solution. In the solid state reactions, equimolar amounts of PbX${}_{2}$ and AX are loaded in a tube, shaken to homogenize the mixture and then heated to obtain a homogeneous solid (in some cases through a melted phase). Alternatively, PbX${}_{2}$ and AX are placed in a mortar and ground with a pestle to get a uniform powder. Finally, multistep synthesis based on the conversion of a seed PbS film, deposited by ALD, into PbX${}_{2}$ and the subsequent formation of the perovskite through a dip in an AX solution has been explored by Sutherland et al. [14]. This procedure yields thin films with highly controlled thickness and excellent purity. Under appropriate synthesis conditions, colloidal lead halide nanocrystals can be obtained in solutions and nanocrystal (NC) films can be deposited onto substrates by means of drop-casting or spin-coating. [15,16].

The first synthesis of metal halide perovskites dates back to the late 1970s [17,18], but their physical and chemical properties have not been deeply investigated until the pioneering research of Miyasaka’s group in 2009 [19], when they used hybrid perovskite as a light absorbing material in a solar cell, reaching less than 4% efficiency. Since then, thanks to their large absorption coefficient, low defect density and high carrier mobility [12,20,21], perovskites have attracted more and more attention, and in a short time all the research efforts have brought to the actual solar cells’ power a conversion efficiency of 25.5% [22].

Together with the development of solar cells, a wide variety of active properties of lead halide perovskites have been demonstrated, such as electroluminescence, efficient photoluminescence and optical gain, thereby showing perovskites to be candidate active materials for other optoelectronic devices.

Metal halide perovskites indeed have also been exploited for the realization of LEDs. Starting from the first reports of the electroluminescence in organic–inorganic perovskites [23,24], lots of advances have been made, leading to bright emission devices [25]. The possibility to deposit perovskite films through solution-based techniques and tune the emission wavelength by varying the chemical composition of the perovskite allowed researches to obtain light emitting diodes with increased efficiency and wide color gamuts [10,26,27,28].

Concurrently, the first report of room temperature amplified spontaneous emission (ASE) has been provided [29], which cleared the path for the implementation of lead halide perovskites as active materials for lasers. Great efforts have been made to improve the ASE properties [16,30,31,32,33,34,35] and lasers have been realized with different geometries, such as microcavity lasers [11], distributed feedback lasers [36], whispering gallery mode lasers [37] and random lasers [38].

Moreover, due to perovskite attractive electrical and optical properties, a new route for the application of perovskites in the detection of high-energy radiation, such as X-ray, gamma and deep-UV photons, has been opened up [39,40,41,42,43,44,45,46,47,48]. In particular, the ability to convert high-energy photons into lower-energy, direct bandgap emissions recently made Cs-based perovskites ideal candidates for implementation in deep-UV photodetectors. As an example, inorganic ${\mathrm{CsPbBr}}_{3}$ quantum dots (QDs) have been integrated into perovskite photodiodes as fluorophors playing two roles: they act as a down-conversion layer, converting incident UV into emitted 510 nm light, and protect hybrid perovskite from UV degradation [46].

However, the lack of long term chemical stability to date still prevents applications in commercial devices.

In particular, many experiments demonstrated that lead halide perovskite films are sensitive to the environment chemical composition, and depending on the chemical nature of the interacting species, the material can show irreversible interactions, leading to degradation or reversible interactions thereby preserving the active properties when the external gas source is removed. The reversible interactions could be potentially exploited for sensing applications.

Another interesting aspect of lead halide perovskites for sensors development is related to their tendency to form films with pores on the surface, potentially allowing an efficient superficial interaction with gas species and improving gas adsorption and evacuation. These features could be important to allow fast sensor response and recovery times and high sensitivity even at room temperature [49].

Recently, various review articles debating the use of perovskite as sensing materials have been published [50,51,52], that deal with different classes of perovskites (such as metal oxide and metal halide perovskites), concern the interaction of the sensing material with metal ions, solvents and gases, and focus on the chemical point of view of the interaction between the perovskite and the analyte [53].

In this paper, differently from what already done, we want to focus on a particular class of perovskites, i.e., lead halide ones, and review the actual state of art of the research on the effects of interaction with the environment. We will initially investigate the main processes inducing perovskite degradation, due to the interaction with light, surrounding atmosphere (oxygen, moisture, ${\mathrm{NH}}_{3}$ or other gases or volatile organic compounds (VOCs)), and due to heating. Then we will focus on the reversible modulation of electrical and optical properties of perovskites, that can be exploited for the development of perovskite gas sensors giving an almost complete overview of the tested devices.

## 2. Environmental Stability of Perovskites

The environmental stability is a particularly relevant property for any material aiming to be used in any application, as it allows the realization of devices with a long operative life. In particular an active material for a solar cell should be stable when operating in air and under sun light, and should also be stable against heating induced by the sunlight exposure. For these reasons, given the impressive increase of the power conversion efficiency of perovskite solar cells, many groups investigated the stability of perovskite thin films under exposure to different external perturbations, with a particular focus on the perovskite irreversible degradation channels.

In this section we will describe the main results in literature about perovskite degradation due to the exposure to light, heating, and environmental species, such as oxygen, moisture or other gases (such as ${\mathrm{NH}}_{3}$, $H_{2}$ and volatile organic compounds).

Even if most of the referenced papers below report stability tests on perovskite for the realization of solar cells, exploring both the variations of structure/morphology and of the electro-optical properties, the results about the intrinsic stability of the material are expected to be of general validity and independent of the specific application.

### 2.1. Light and Temperature Effects

Most of the experiments about lead halide perovskites have been performed on iodide perovskites, and in particular, on ${\mathrm{MAPbI}}_{3}$, which thanks to its absorption edge in the near infrared, has been widely used as active material in solar cells.

A preliminary interesting result on the ${\mathrm{MAPbI}}_{3}$ stability has been obtained on active films stored in dark and vacuum condition [54], surprisingly demonstrating that the material shows irreversible degradation even if not exposed to light and to external chemical species. The self degradation was ascribed to a sequence of four different processes: decomposition of ${\mathrm{MAPbI}}_{3}$ in ${\left[\mathrm{MA}\right]}^{+}$, ${\left[{\mathrm{PbI}}_{2}\right]}^{-}$ and ${\left[I\right]}^{-}$ ions; dissociation of ${\left[{\mathrm{PbI}}_{2}\right]}^{-}$ into ${\mathrm{Pb}}^{0}$ and $I_{2}^{-}$ at the perovskite surface; regeneration of $I_{2}^{-}$ ions at the perovskite interface and formation of AuI${}_{2}$ at the interface with the gold electrode of the solar cell device.

Starting from this evidence of intrinsic instability it is easy to understand that additional factors, such as light exposure, heating or interaction with the environment can induce further degradation processes.

**Figure 1:**
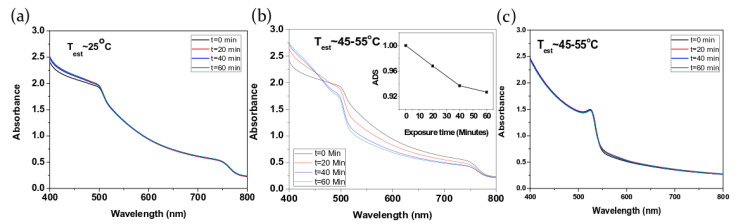
UV–Vis absorption spectra of encapsulated ${\mathrm{MAPbI}}_{3}$ films exposed for various times at (**a**) ≃25 ${}^{{}^{\circ}}$C and (**b**) ≃45–55 ${}^{{}^{\circ}}$C: spectral modifications appear at high temperature, whereas (**c**) the ${\mathrm{MAPbBr}}_{3}$ film remains stable even at ≃45–55 ${}^{{}^{\circ}}$C. Adapted with permission from [55]. Copyright © 2015, American Chemical Society.

The effects of sunlight exposition are reported by Misra et al. [55] who investigated the material stability by measuring the evolution of the absorption spectra of ${\mathrm{MAPbI}}_{3}$ thin films, deposited and encapsulated in a glove box (thus exposed to light in an inert environment), and exposed to concentrated sunlight of 100 suns. The authors observed that light exposure for times up to 1 h has no effects on the absorption spectra if the sample was kept at 25 ${}^{{}^{\circ}}$C (see Figure 1a). On the contrary, a clear variation of the absorption is observed when the film is exposed to concentrated sunlight with a film temperature increase to only 45–55 ${}^{{}^{\circ}}$C (see Figure 1b). In particular, beyond a general reduction of the total absorption (see inset of Figure 1b), a clear variation of the absorption lineshape is observed, with a reduction of the intrinsic ${\mathrm{MAPbI}}_{3}$ absorption and an increase of absorption in the range 400–460 nm, ascribed to ${\mathrm{MAPbI}}_{3}$ decomposition with crystallization of its inorganic component ${\mathrm{PbI}}_{2}$. This effect is not observed when the sample is heated at the same temperature without light exposure, evidencing that a combination of heating and lighting is required to determine the degradation. In the same experiment the authors also demonstrated that no degradation takes place in ${\mathrm{MAPbBr}}_{3}$ films, both at 25 ${}^{{}^{\circ}}$C and at 45–55 ${}^{{}^{\circ}}$C, clearly evidencing that the light-induced degradation is highly dependent on the hybrid perovskite composition (see Figure 1c). The higher stability of Br-based hybrid lead halide perovskite with respect to the I-based one was ascribed to differences in the ionic ratio of ${\mathrm{Br}}^{-}$ and $I^{-}$ ions that influences the perovskite crystal structure. In particular the cubic crystalline phase of ${\mathrm{MAPbBr}}_{3}$ results denser and thus less prone to environmental molecules attacks than the tetragonal structure of ${\mathrm{MAPbI}}_{3}$.

The higher stability of ${\mathrm{MAPbBr}}_{3}$ with respect to ${\mathrm{MAPbI}}_{3}$ also comes from the higher bromine electronegativity and to the stronger Pb-Br bond compared to the Pb-I one, and of the H-Br bond compared to the H-I one [54,55].

The details of photo-degradation and thermal decomposition of ${\mathrm{MAPbI}}_{3}$ and ${\mathrm{MAPbBr}}_{3}$ have been also investigated by Juarez-Perez [56]. Beyond evidencing that light and heat can, in proper conditions, determine the perovskite degradation, the experiment allowed to conclude that parts of the reaction determining the release of gas compounds are reversible and cannot be considered real degradation channels. In particular the released ${\mathrm{CH}}_{3}{\mathrm{NH}}_{2}$ cannot be considered as degradation products of perovskites because they can resynthesize ${\mathrm{MAPbI}}_{3}$ if the film is in a closed environment (for example, an encapsulated film). On the contrary, the back formation to MAX (X = Br, I) or ${\mathrm{MAPbX}}_{3}$ from the released ${\mathrm{CH}}_{3}X$ + ${\mathrm{NH}}_{3}$ molecules is thermodynamically unfavorable and prone to form non-primary ammonium salts, and it is thus an authentic detrimental pathway for perovskite degradation.

Further insight in the light induced ${\mathrm{MAPbI}}_{3}$ degradation processes was obtained by X-ray photoelectron spectroscopy (XPS) on films irradiated in vacuum by a blue laser [57]. A two step degradation process was proposed, with an initial decomposition of ${\mathrm{MAPbI}}_{3}$ leading to the formation of ${\mathrm{PbI}}_{2}$ and volatile compounds, such as ${\mathrm{NH}}_{3}$ and HI. Then, ${\mathrm{PbI}}_{2}$ further decomposes into metallic lead (${\mathrm{Pb}}^{0}$) and iodine ($I_{2}$). A saturation of the degradation was observed after 480 min of laser irradiation, when the ratio of metallic Pb to total Pb was about 33%.

The thermal stability of ${\mathrm{MAPbI}}_{3}$ perovskite films has been also investigated by Conings et al. [58] in various ambient conditions (pure $N_{2},O_{2}$ and ambient air), corroborating the idea that bringing perovskite films to a temperature of 85 ${}^{{}^{\circ}}$C, which is close to the temperature at which perovskite is formed, can induce surface decomposition, even in an inert atmosphere. The presence of oxygen and water molecules further accelerates the disintegration process since they react with the methylammonium iodide (MAI) cation, leading to the breakage of the perovskite unit cell.

A nice example of the correlation between the active material degradation and the variation of its optical properties has been obtained by Motti et al. [59] who analyzed the effects of the photoexcitation energy density on the photoluminescence (PL) and the transient absorption of polycrystalline thin films, of both hybrid (${\mathrm{MAPbBr}}_{3}$ and ${\mathrm{MAPbI}}_{3}$) and fully inorganic (${\mathrm{CsPbBr}}_{3}$) perovskites.

**Figure 2:**
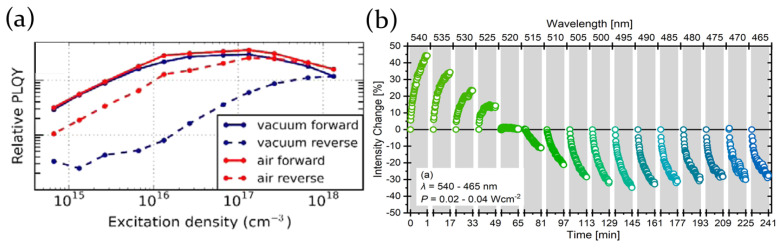
(**a**) Relative photoluminescence quantum yield (PLQY) curves of ${\mathrm{MAPbBr}}_{3}$ polycrystalline thin films in air (red) and in a vacuum (blue). Adapted with permission from [59]. Copyright © 2016, American Chemical Society. (**b**) Percentage PL intensity change of a ${\mathrm{MAPbI}}_{3}$ film for 60 s of illumination in air, for excitation wavelength between 540 and 465 nm (gray shades). Between each measurement the sample was kept for 15 min in the dark in order to reduce the overall stress (white columns); the emission was detected at the PL peak position (778 nm). Adapted with permission from [60]. Copyright © 2018, American Chemical Society.

In all the investigated materials they demonstrated that the laser pumping results in the formation, even in vacuum, of sub-bandgap defect states, quenching the PL. This effect is evidenced by PL measurements as a function of the excitation density showing a progressive increase of the PL intensity as the excitation density increases, but also a systematically lower PL intensity when the excitation density is decreased back to the initial value (see Figure 2a). This effect is also present when the samples are pumped in air, but the relative decrease of the PL intensity is, surprisingly, much weaker. This suggests that the presence of oxygen in the atmosphere, even in small amounts, attenuates the effect as it acts as passivating agent for such defect states.

The role of the excitation wavelength on ${\mathrm{MAPbI}}_{3}$ perovskite thin films has instead been described by Quitsch et al. [60]. They demonstrated that an increase of the photoluminescence intensity (called photobrightening) occurs under green laser illumination (λ > 520 nm), while the decrease of the emitted PL is obtained by excitation with a blue led (λ < 520 nm), as shown in Figure 2b. In particular, the threshold wavelength, separating photobrigthening from photodegradation, is determined by the ${\mathrm{PbI}}_{2}$ energy band gap. The quenching of the photoluminescence is indeed related to a photodegradation process, ascribed to the formation of $I_{2}$ by photolysis of ${\mathrm{PbI}}_{2}$ and/or hole transfer between the photoexcited species under blue light exposure. Moreover, similarly to the previous report, oxygen has been demonstrated to have a positive role in inducing the photobrightening, since oxygen-based species act as passivating agents for perovskite halide vacancies.

The evidence of several degradation channels in hybrid organic–inorganic perovskites stimulated the research of novel chemical compositions allowing a stability increase. In this frame a particularly interesting class of materials are fully inorganic perovskites, in which the organic cation is replaced by an inorganic atom (typically Cs) [61].

A couple of interesting experiments have been performed in order to investigate the stability of ${\mathrm{CsPbBr}}_{3}$ perovskite nanocrystals films [62,63], evidencing that also these materials show several degradation processes induced by light and depending on the environment chemical composition.

In particular it has been shown that under illumination of 450 nm light-emitting diode they suffer degradation, resulting in a sample color change from green to yellow, both in solutions and in films.

**Figure 3:**
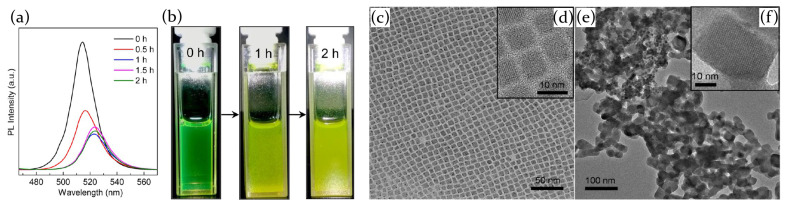
(**a**) PL emission spectra of the colloidal ${\mathrm{CsPbBr}}_{3}$ toluene solution and (**b**) the optical images of the cuvette as a function of the illumination time ($175\;\mathrm{mW}/{\mathrm{cm}}^{2}$). (**c**) Transmission electron microscope (TEM) and (**d**) high resolution transmission electron microscope (HRTEM) images of ${\mathrm{CsPbBr}}_{3}$-NCs. (**e**) TEM and (**f**) HRTEM images of the a ${\mathrm{CsPbBr}}_{3}$ sample after 2 h of illumination. Adapted with permission from [62]. Copyright © 2017, American Chemical Society.

As highlighted in Figure 3c–f, the pristine green nanocrystal (NC) solution is formed by highly monodispersed nanocrystals with an average length of about 5–10 nm; as the exposition time goes on, NCs tend to aggregate forming clusters, up to 50 nm after about 2 h of illumination, and the solution color turns to yellow (Figure 3a,b). This effect has been observed both in solution and in films and is ascribed to the light source which removes bonding ligands from the surface of the nanocrystals, allowing them to merge and form bigger aggregates. Concurrently, the PL emission intensity decreases as a function of the illumination time, coming with a redshift of the PL peak. The surface decomposition of the nanocrystals determines indeed an increase of the surface charge trap states, which quenches the sample light emission, whereas the shift toward longer wavelengths is attributed to the greater crystals dimensions. Moreover, the degradation process has been demonstrated to be dependent on the power illumination, since a 89.4% PL loss has been obtained at $175\;\mathrm{mW}/{\mathrm{cm}}^{2}$ and it reaches higher values (99.3%) at $350\;\mathrm{mW}/{\mathrm{cm}}^{2}$ for ${\mathrm{CsPbBr}}_{3}$ films.

In addition to illumination, other factors have been reported to influence the sample degradation. The color variation is indeed not present in encapsulated samples, evidencing the effects is not simply due to photodegradation. On the other hand, no degradation is also observed if the samples are stored in dark, even in oxygen atmosphere, suggesting that a combination of light exposure and oxygen is necessary to induce the degradation.

The role on the NCs size for ${\mathrm{CsPbBr}}_{3}$ films under UV light illumination has also been investigated in [63], evidencing that the morphology of the film remains more stable (quite unchanged) for samples composed of bigger grains. The authors deposited two ${\mathrm{CsPbBr}}_{3}$ NCs films with an average crystal dimensions of 9.7 nm and 11.4 nm, respectively, emitting at 495 nm (NC495) and 520 nm (NC520). The experimental results evidenced that under UV light exposure NC520 NCs remain rather unchanged, whereas smaller crystals in the NC495 sample tend to merge and form bigger oval-shaped aggregates, related to the formation of ${\mathrm{Cs}}_{4}{\mathrm{PbBr}}_{6}$ products, as evidenced in XRD spectra.

However, both samples (NC495 and NC520) suffer from the combined detrimental action of light irradiation and temperature, since both show a PL intensity decrease for increasing temperature values from 80 K to 400 K, under UV light illumination. The observed quenching of the photoluminescence is probably related to the formation of trap states which limit radiative recombination processes, and are ascribed to the photooxidation mechanism of lead atoms in perovskite films.

### 2.2. Air, Oxygen and Humidity Effects

There is strong evidence that in presence of air, oxygen and/or humidity the perovskites can modify their properties, and the irradiation and temperature effects are enhanced. With the aim to understand the ambient induced degradation, numerous investigations have been carried on by studying the effects of oxygen and wet or dry air.

For example, close attention has been payed to the role of humidity in the atmosphere in contact with a perovskite sample, even if opposing results have been reported. Some of them point out a detrimental action of water molecules on the morphological stability of the perovskite structure, others evidence that it does not inevitably lead to deleterious effects.

The ascertained intrinsic thermal instability of ${\mathrm{MAPbI}}_{3}$ [58] is boosted in ambient air. Even if the samples annealed at 85 ${}^{{}^{\circ}}$C after 24 h show structure variation regardless the atmosphere ($N_{2}$, dry $O_{2}$ or ambient air), the authors found that the major changes are induced when the samples are treated in air. Water and oxygen molecules can interact with the methylammonium cations, leading to the formation of new carbon based species and the release of nitrogen. Moreover, moisture accelerates the formation of metallic lead (${\mathrm{Pb}}^{0}$) clusters from ${\mathrm{Pb}}^{2+}$ ions, in addition to the most commonly known formation of ${\mathrm{PbI}}_{2}$ as degradation product of ${\mathrm{MAPbI}}_{3}$ perovskite.

**Figure 4:**
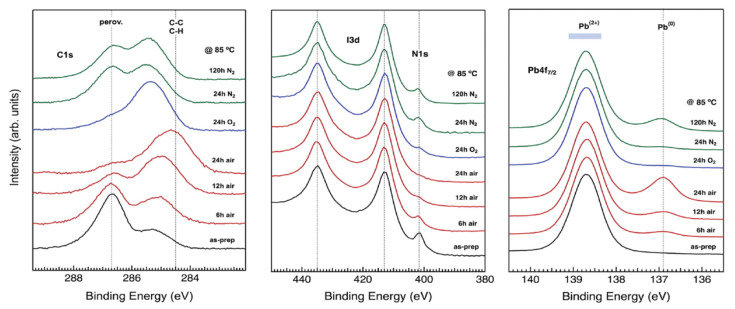
XPS measurements on ${\mathrm{MAPbI}}_{3}$ perovskite samples treated at 85 ${}^{{}^{\circ}}$C for up to 24 h in different atmospheres. Adapted with permission from [58]. Copyright © 2015, WILEY-VCH Verlag GmbH and Co. KGaA, Weinheim.

All these degradation pathways find confirmation in XPS measurements, as reported in Figure 4. The left panel of Figure 4 shows the carbon compounds XPS peaks of the as-prepared sample (black curve) and for the sample stored in different atmospheric conditions (air, oxygen and nitrogen, up to 120 h of storage). The pristine sample is characterized by two carbon contributions, one at higher binding energy (286.7 eV) and the other one at lower binding energy (285.3 eV). The first one is ascribed to the C-N bound of the MAI cation (indicated as “perov.” in the figure), whereas the second one refers to C-C and C-H bounds of hydrocarbons formed on the perovskite surface at the deposition stage.

Heating the sample supports the release of the methylammonium volatile compounds, leading to a gradual disappearance of the C-N bound peak, in favor of the hydrocarbon ones. This effect results to be more prominent in presence of oxygen and water molecules, and is corroborated by a concurrent decrease of the N1s core level peak intensity, at 402 eV, sign of the release of $N_{2}$ molecules from the perovskite surface (central panel of Figure 4). Besides, the panel on the right evidences the appearance of metallic lead with the ${\mathrm{Pb}}^{0}$ peak at about 137 eV at increasing exposition times in air.

Humidity is also revealed to be a critical factor in the performance of ${\mathrm{MAPbI}}_{3}$ solar cells due to the deleterious effects of $H_{2}O$ vapor on the perovskite [64]. Morphology and crystal structure measurements have been performed for perovskite films exposed to different atmospheric conditions in the dark (0–90% RH). It has been observed that before humidity exposure all the perovskite samples are characterized by a rough surface whereas, after being in contact with water molecules, they undergo a recrystallization process, becoming smooth and highly ordered; moreover, this process is more emphasized for higher humidity content. X-ray diffraction (XRD) measurements then prove the formation of an hydrate product, similar to ${\left({\mathrm{CH}}_{3}{\mathrm{NH}}_{3}\right)}_{4}{\mathrm{PbI}}_{6}\cdot 2H_{2}O$, after the exposure of perovskite to humid air in the dark. Anyway, the hydration process seems to be reversible and the perovskite can partially recover its pristine structure after being stored in vacuum or dry atmosphere. In addition, time-resolved absorption spectra highlighted that there are not radical changes in perovskite typical peaks for samples stored up to 14 days in different humidity conditions meaning that, despite the morphological changes observed, the perovskite excited state properties do not change.

**Figure 5:**
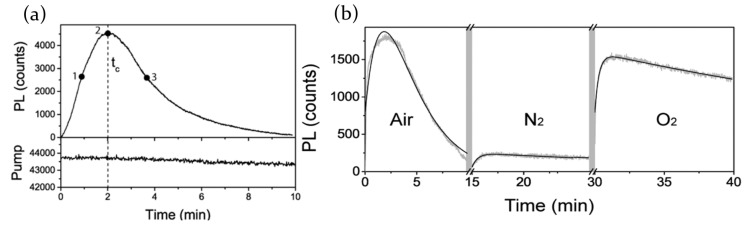
(**a**) Temporal evolution of PL intensity maximum at 775 nm for ${\mathrm{MAPbI}}_{3}$ perovskite films, under monochromatic light excitation (500 nm), showing an initial photoactivation stage followed by intensity quenching after $t_{c}$. (**b**) Evolution of the PL peak intensity when switching the atmosphere from air to nitrogen and then to pure oxygen. Adapted with permission from [65]. Copyright © 2015, American Chemical Society. https://pubs.acs.org/doi/10.1021/acs.jpclett.5b00785. Further permission should be directed to the ACS.

In agreement with the previous report, Galisteo-Lopez et al. [65] rule out the irreversible detrimental action of moisture on ${\mathrm{MAPbI}}_{3}$ perovskite film chemical composition. Optically pumping the sample, the PL maximum registered at 775 nm shows an initial photoactivation stage followed by a radiation quenching (Figure 5a). Measurements taken in different atmospheres (ambient air, $N_{2}$ and $O_{2}$; Figure 5b) allow one to identify moisture and oxygen as the responsible molecules for photodarkening and photoactivation processes, respectively. Water molecules, in particular, induce the formation of hydrates species on the surface of perovskite, determining a quenching of the photoluminescence. Switching the atmosphere from ambient air to a pure oxygen one allows, on the other hand, to partially recover the sample emission, evidencing that $O_{2}$ acts as a trap states filler.

Even if the presence of moisture in the atmosphere surrounding the sample influences its photoluminescence emission, it has been verified that water molecules do not cause permanent degradation of the film. Under prolonged optical pumping in humid conditions, it has been indeed demonstrated that the film does not suffer any color change in its aspect, proving that the interaction of perovskite with water molecules does not induce the formation of ${\mathrm{PbI}}_{2}$ molecules on the sample surface.

On the contrary, other works support the idea of moisture induced permanent degradation, both in ${\mathrm{MAPbI}}_{3}$ [66,67], ${\mathrm{FAPbI}}_{3}$ [68] and mixed halide perovskites [69].

Concerning the interaction between the active mean and the environment (in particular, moisture), film morphology seems to play a fundamental role. Being a phenomenon related to uppermost part (surface) of the perovskite, the morphology and the dimensions of the grains composing the deposited film, the presence of grain boundaries and their chemical composition can deeply influence the interaction process, and as a consequence, limit or accelerate the degradation of the film.

The morphology of ${\mathrm{FAPbI}}_{3}$ perovskite films, for example, has been analyzed through atomic force microscope (AFM) topography images, which evidenced that the sample remains stable when stored in controlled atmosphere below 30% of relative humidity (RH) at room temperature, but suffers moisture action when the atmosphere reaches 50% RH, and completely decomposes in ${\mathrm{PbI}}_{2}$ when soaked in water.

**Figure 6:**
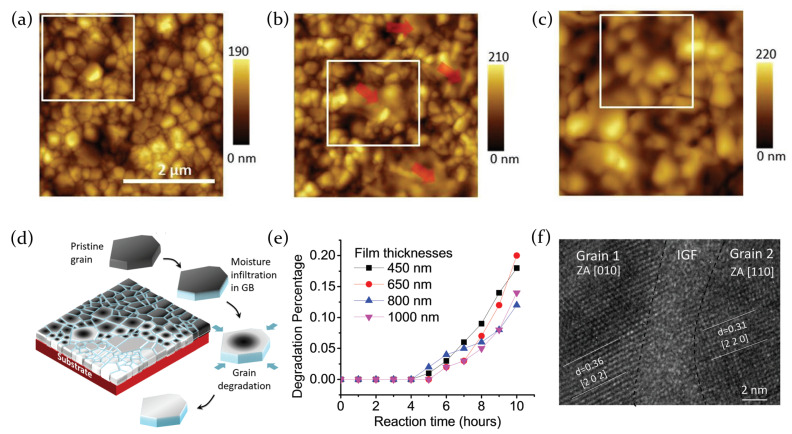
(**a**–**c**) AFM images of ${\mathrm{FAPbI}}_{3}$ film, showing the different stages of the degradation process, from the fresh sample (dark black film) to the degraded one (pale yellow). Adapted with permission from [68]. Copyright 2018, WILEY-VCH Verlag GmbH and Co. KGaA, Weinheim. (**d**) Schematic representation of the degradation of ${\mathrm{MAPbI}}_{3}$ perovskite film in moisture; the degradation mechanism starts from grain boundaries and extends towards the grain center along the in-plane direction. (**e**) Degradation percentage as a function of the moisture exposure time for different film thicknesses. (**f**) A HRTEM cross-sectional image highlighting the presence of an intergranular film (IGF) between two neighboring grains. Republished with permission of the Royal Society of Chemistry, from [67], © 2017.

In particular, grain boundaries result to be the starting point for perovskite decomposition, representing a pathway for the interaction with water molecules; the degradation then proceeds deeper into the grains. Once the degradation process is started, the morphology of the film changes, resulting in a merging of neighbour grains which form bigger clusters and the appearance of a non perovskite phase, as evidenced in Figure 6a–c [68].

As a consequence, a scaling behavior with grain size has been revealed [67]: since degradation process starts from grain boundaries (Figure 6d), the perovskite films composed of bigger grains result more stable than the films with smaller grains. Another peculiarity relates to the fact that the degradation process, starting from grain boundaries, expands along in-plane direction when the sample is annealed after deposition; otherwise, when the sample does not receive any post-deposition treatment, chemical modifications result more uniform. An annealing process, indeed, facilitate the release of MA ions or MAI from the surface of the perovskite, leaving a ${\mathrm{PbI}}_{2}$-terminated surface. As ${\mathrm{PbI}}_{2}$ is less soluble in water than MA ions, the top plane surface of the annealed film results less prone to humidity degradation. Moreover, grain boundaries have been seen to be composed of an amorphous region, caused by an excess of MAI during the film formation phase, which facilitates moisture permeation and then induces degradation (Figure 6f).

The chemical composition of the active material has been proved to play a fundamental role in the response to humidity, as evidenced through the experiment of Howard et al. [69] on thin films of ${\mathrm{Cs}}_{x}{\mathrm{FA}}_{1-x}\mathrm{Pb}{\left(I_{y}{\mathrm{Br}}_{1-y}\right)}_{3}$ mixed halide perovskites. Different concentrations of Cs/FA cations and I/Br halides have been chosen in order to show which is the photoluminescence response of the films at different humidity concentrations (with RH ranging from 5 to 55%).

At low humidity levels, for RH ranging from 5% to 35%, all the samples analyzed, regardless their chemical composition, show an enhancement of the emitted light intensity for increasing humidity content. Water molecules, indeed, remove defect states not already passivated from $O_{2}$ molecules, determining a PL intensity increase.

Switching the atmosphere from 35% to 55% of RH, on the other hand, the chemical composition of the active material results to be crucial. Low (17%) Br content samples, indeed, maintain the PL intensity at the same level; high (38%) Br content samples, instead, show a PL intensity decrease combined with a PL peak red-shift, both independently from the Cs percentage.

The presence of high humidity content, moreover, is responsible for the degradation of the perovskite. 55% of RH is indeed sufficient to form a thin layer of monohydrate species on the perovskite surface. As the water adsorption proceeds, dihydrate species are formed, leading to perovskite degradation with ${\mathrm{PbI}}_{2}$, ${\mathrm{PbBr}}_{2}$ and ${\mathrm{Cs}}_{x}{\mathrm{FA}}_{1-x}I_{y}{\mathrm{Br}}_{1-y}$ formation and the presence of new surface trap states. Since moisture presence influences the halide ions mobility in the perovskite crystal structure, major Br-content samples results to be more affected by degradation and suffers PL intensity quenching processes to a greater extent.

Another chemical species deeply investigated is oxygen; metal halide perovskites have been revealed indeed to be highly sensitive to the presence of O${}_{2}$ molecules, both in a pure oxygen atmosphere and in ambient air.

Fang et al. [70], for example, showed how the atmosphere surrounding a ${\mathrm{MAPbBr}}_{3}$ perovskite single crystal can modulate its photoluminescence emission. Pumping the sample with a 400-nm excitation laser, with a pulse repetition of 1.4MHz, the effect of different gases has been studied, monitoring the PL emission every 10 s. Is has been verified that the presence of dry $N_{2}$, ${\mathrm{CO}}_{2}$ and Ar does not affect perovskite light emission in time; air, dry $O_{2}$ and wet $N_{2}$ instead induce PL changes, in particular determining photoactivation processes. The major PL enhancement is observed in presence of air, suggesting that oxygen and water molecules are the real responsible for the observed emission variations. Switching the chamber atmosphere to vacuum condition, an almost immediate photoluminescence quenching is observed, and the process results to be reversible when going back to air atmosphere.

The experimental results thus lead to think that the interaction between perovskite and water/oxygen molecules is a physisorption, rather than a chemisorption. Oxygen and water molecules role is to act as electron donors, neutralizing an excess of positive ${\mathrm{Pb}}^{2+}$ charges on perovskite. Since those trap states have been demonstrated to be located mostly on the surface rather than in the perovskite bulk, a control of the surface trap states density through sample exposure to different gases is possible. As a consequence, a reversible PL intensity modulation can be obtained and ${\mathrm{MAPbBr}}_{3}$ single crystals become suitable for new potential applications in the detection of oxygen and water vapor.

Similarly to what reported above, also Zhang et al. [71] observed a PL intensity modulation from ${\mathrm{MAPbBr}}_{3}$ perovskite single crystals. Interestingly, the analysis evidenced that the photoluminescence intensity decrease, obtained passing from air to vacuum atmosphere, was accompanied by a corresponding modulation of the electrical properties. In particular, both dark current and photocurrent, measured through the gold electrodes deposited on the ${\mathrm{MAPbBr}}_{3}$ film surface, have been observed to increase in vacuum conditions. This suggests the formation of shallow trap states in ambient air exposure; since those trap states are close to the perovskite band edges, photogenerated carriers can be trapped in, forming radiative recombination centers and then leading to a PL enhancement. As a consequence, in presence of air charge carriers are trapped and radiatively recombine, and can not be available for photocurrent in the perovskite device; viceversa, in vacuum conditions, the density of trap states is reduced and the current increases.

Air, moisture and light stimuli have then been also studied together, in order to evaluate the possible synergistic effects arising from their combined presence.

As an example, Tian and its coworkers [72] investigated the luminescence properties of a spin coated ${\mathrm{MAPbI}}_{3}$ perovskite polycrystalline film, studying both the PL intensity variation by CW 514 nm excitation laser and the PL decay dynamics under picosecond pulsed laser, when the sample is exposed to different external stimuli. In particular, they focused on the curing action induced by the combined presence of light and oxygen, needed to deactivate quenching sites in the perovskite crystal structure. As a consequence, initial low PL intensity can increase for more than three order of magnitudes as the light exposition goes on. Along with the PL intensity enhancement, also a PL lifetime increase, from several nanoseconds to several hundreds of nanoseconds, has been observed. Excitons and free carriers (electrons and holes) created from the absorption of photons can be trapped by perovskite defect sites, thereby inducing non radiative recombinations. The combined presence of oxygen and light can, however, reduce the concentration of trapping sites and increase the charge carrier diffusion length. As a consequence, if initially the process is confined to the upper region of the perovskite layer, as the curing action goes on, trapping sites in bulk perovskite can be filled.

Other works confirming the competing action of photobrightening and photodarkening are reported in literature.

As an example, Godding et al. [73] used ${\mathrm{MAPbI}}_{3}$ films excited with a picosecond pulsed laser emitting at 504 nm, in order to explore the effects of the combined action of light excitation and air exposure on the photoluminescence properties of the sample. In particular, they observed an initial photobrightening phase, followed by a worsening stage of the emission properties in which the PL intensity starts to saturate. Light excitation is thought to determine the modification of the perovskite stoichiometry, through the generation of atomic lead and the loss of iodine, increasing surface state density responsible for charge non-radiative recombination. Molecular oxygen then diffuses into the film forming superoxide species ($O_{2}^{-}$), whose interaction with methylammonium cations leads to peroxide $H_{2}O_{2}$ formation. The latter, in turn, interacts with atomic lead, forming lead oxide (PbO). This oxidation process improves the emission properties since it contributes to passivate trapping sites. On the other hand, the simultaneous loss of MA ions and the formation of ${\mathrm{PbI}}_{2}$ overcomes the beneficial effect of passivation and brings to perovskite degradation.

Motti et al. [59] repeated the study of the irradiation effect on perovskite polycrystalline films in atmosphere and showed that PL emission greatly increases when passing from vacuum to air. Comparison between the results obtained in wet and in dry air leads to conclude that oxygen is the molecule responsible of the observed PL enhancement. As stated above, light irradiation creates sub band-gap states which trap carrier charges and induce an instant PL quenching. Filling the chamber with air, however, allows one to restore the photoluminescence, thanks to the combined action of light and oxygen, which passivates defects. PL decay and transient absorption (TA) measurements, moreover, allow one to shed light on the PL decay dynamics, showing the presence of a fast component, on the order of few nanoseconds, and a slow one, which extends on microsecond time scale. The first one is associated with the carrier trapping mechanism, whereas the second one is related to the recombination of trapped carriers. Exposing the sample to oxygen reduces the trap sites density; as a consequence, the fast decay component shows a longer lifetime compared to inert atmosphere, whereas the slow component almost disappears.

Starting from the first evidence of the effect of the excitation wavelength on ${\mathrm{MAPbI}}_{3}$ perovskite thin films (which showed photobrightening induced by green excitation light and photodegradation by blue light), Quitsch et al. [60] further went into the issue in order to reveal which is the role of the atmosphere. For this purpose, photostability measurements have been performed both in vacuum and in ambient air.

The experimental results evidenced that under green laser illumination a photobrightening effect is always observed and appears to be promoted in air. On the other hand, the presence or absence of humidity in the atmosphere reveals to be crucial for a blue light illumination. In vacuum or dry air condition, indeed, the PL intensity initially increases and then suffers a decrease with ongoing illumination time, sign of the degradation of the material. Differently, in presence of humid atmosphere, the photodegradation process, responsible for the PL quenching, is boosted and completely hides the photobrightening effect. These results suggest the coexistence of a wavelength-independent photobrightening and a wavelength-dependent photodegradation effect in ${\mathrm{MAPbI}}_{3}$ upon excitation above ${\mathrm{PbI}}_{2}$ band gap.

According to the authors, the reason behind the increase of the photoluminescence is attributable to the passivation of iodine vacancies on the perovskite surface. These vacancies indeed act as non-radiative trap states located in energy immediately below the conduction band minimum. Once they are passivated (for example, by superoxide ions formed due to the presence of light and oxygen), sub-bandgap states shift into the valence band and trap states density is reduced, determining an increase of the PL intensity [74].

On the other hand, exciting the sample with a wavelength shorter than 520 nm allows the formation of ${\mathrm{PbI}}_{2}$, detrimental for the stability of the perovskite, which therefore brings to a PL decrease.

The presence of a light source, combined with an oxygen rich atmosphere, is thought to be responsible for the formation of superoxide species ($O_{2}^{-}$) through charge transfer from perovskite to molecular oxygen, thereby activating degradation processes of the active mean.

**Figure 7:**
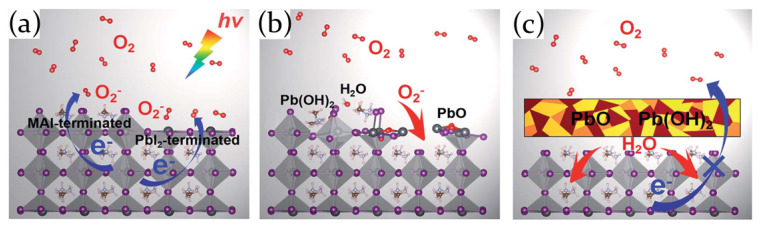
Schematic representation of the photo-oxidative degradation process of the ${\mathrm{MAPbI}}_{3}$ surface. (**a**) $O_{2}$ molecules close to the surface of ${\mathrm{MAPbI}}_{3}$ get the photo-excited electrons from perovskite, forming superoxide anions ($O_{2}^{-}$). (**b**) $O_{2}^{-}$ reacts with ${\mathrm{MA}}^{+}$ and Pb, yielding $H_{2}O$ and $\mathrm{Pb}{\left(\mathrm{OH}\right)}_{2}$ on the MAI-terminated surface. Oxidation of lead on the ${\mathrm{PbI}}_{2}$-terminated surface leads to the disintegration of the Pb–I bond, producing PbO and leaving the underlying MAI-terminated surface exposed. (**c**) The oxidation products PbO and $\mathrm{Pb}{\left(\mathrm{OH}\right)}_{2}$ form a protection layer, preventing the oxidation of inner ${\mathrm{MAPbI}}_{3}$. However, the fresh water molecules produced in previous steps cause hydration of the inner perovskite. Used with permission from the Royal Society of Chemistry from [75], © 2019; permission conveyed through Copyright Clearance Center, Inc.

The photo-oxidative degradation mechanism has been hypothesized by Ouyang et al. [75] for ${\mathrm{MAPbI}}_{3}$ perovskite and includes three steps, as schematically represented in Figure 7a–c.

First, superoxide anions ($O_{2}^{-}$) are created through a charge transfer from perovskite surface to molecular oxygen. The second step depends on the surface composition; indeed, since ${\mathrm{MAPbI}}_{3}$ perovskite surface can be ${\mathrm{PbI}}_{2}$-terminated or MAI-terminated, two different degradation mechanisms are proposed. For a ${\mathrm{PbI}}_{2}$-terminated surface, due to the higher electronegativity of oxygen compared to iodine, oxygen atoms replace I atoms on the surface, forming Pb-O bonds. The formation of lead oxide results in the release of iodine molecules ($I_{2}$), with a consequent breakage of the surface and the unveiling of the MAI-terminated layer. The exposed MAI-terminated surface is then further oxidized and PbO, $H_{2}O$, and the unstable $\mathrm{Pb}{\left(\mathrm{OH}\right)}_{2}$ are formed. Third, the oxidation products PbO and $\mathrm{Pb}{\left(\mathrm{OH}\right)}_{2}$ form a protection layer to prevent a further oxidation of inner perovskite. Nevertheless, the fresh $H_{2}O$ molecules produced in the previous steps act as active species, causing hydration of the inner perovskite and ultimately destroying the ${\mathrm{MAPbI}}_{3}$.

**Figure 8:**
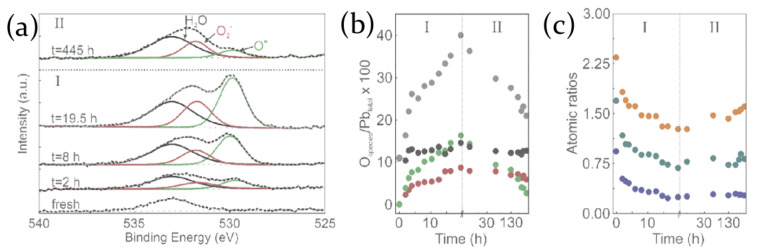
(**a**) Photoelectron energy spectra for (**I**) a photoexcited ${\mathrm{MAPbBr}}_{3}$ sample at different times and (**II**) after keeping the sample stored in the dark and in vacuum, with a deconvolution of different oxygen signals. (**b**) Evolution of the $O_{\mathrm{total}}/{\mathrm{Pb}}_{\mathrm{total}}$ (gray circles), $H_{2}O/{\mathrm{Pb}}_{\mathrm{total}}$ (black circles), $O_{2}^{-}/{\mathrm{Pb}}_{\mathrm{total}}$ (red circles) and $O^{2-}/{\mathrm{Pb}}_{\mathrm{total}}$ (green circles) atomic ratios for the whole experiment, for phases I and II. (**c**) Evolution of the Br/Pb (orange circles), C/Pb (dark green circles) and N/Pb (blue circles) atomic ratios for the whole experiment, for phases I and II. Adapted with permission from [76]. Copyright © 2018, American Chemical Society. https://pubs.acs.org/doi/10.1021/acs.jpclett.8b01830. Further permission should be directed to the ACS.

The formation of negatively charged oxygen molecules influencing the PL properties of the sample is reported also in [76]. The negative oxygen molecules form a layer on the perovskite surface activating, by electrostatic repulsion, the migration of interstitial halide anions toward the perovskite bulk. This mechanism favors the formation of halide vacancy/interstitial Frenkel pairs reducing the density of non-radiative trap states in the bulk of the material and thus inducing the increase of the PL intensity. On the other hand, light exposition in an oxygen atmosphere concurrently induces ${\mathrm{Pb}}^{0}$, ${\mathrm{Br}}_{2}$ and methylammine formation, leading to ${\mathrm{MAPbBr}}_{3}$ degradation and PL decrease. When light and oxygen sources are removed, oxygen species ($O_{2}^{-}$ and water molecules, in particular) remain on the perovskite surface, as inferred from XPS measurements; Br signal is partially recovered, but C and N are irreversibly lost, confirming the hypothesis of volatile methylammine formation and the disintegration of the perovskite lattice (Figure 8a–c) [76].

A combination of experimental measurements and theoretical density fuctional theory (DFT) calculations allowed Aristidou et al. [77,78] to evaluate the effect of the exposure of perovskite films to oxygen and identify the route of superoxide species formation. The authors focused their attention on a couple of samples, namely ${\mathrm{MAPbI}}_{3}$ and ${\mathrm{MAPbI}}_{3}\left(\mathrm{Cl}\right)$, which are identical in chemical composition but differ for the deposition stage. The ${\mathrm{MAPbI}}_{3}\left(\mathrm{Cl}\right)$ has been synthesized by using chloride as precursor, even if it does not influence the final chemical composition of the film. Chlorine ions in the precursor mixture is known to slow down the perovskite formation process, allowing to obtain grains bigger than those formed in ${\mathrm{MAPbI}}_{3}$.

First of all, the oxygen diffusion mechanism in ${\mathrm{MAPbI}}_{3}$ and ${\mathrm{MAPbI}}_{3}\left(\mathrm{Cl}\right)$ perovskite films has been analyzed with isothermal gravimetric analysis (IGA) and time of flight-secondary ion mass spectrometry (ToF-SIMS) measurements, evidencing that the diffusion of $O_{2}$ into the lattice is rapid, reaching the saturation after only 5–10 min, and this represents the reason of the high instability of the material. Then they analyzed through DFT simulations the combined effect of light illumination and oxygen presence, leading to perovskite degradation. Iodine vacancies are formed under light illumination and act as non radiative recombination centers; then, due to an electron transfer mechanism, $O_{2}^{-}$ ions are formed. The entire mechanism is shown to be influenced by the particle size of the film: smaller grains show higher defect density and thus, minor stability, whereas films composed of larger crystals result to be less prone to degradation. As a consequence, ${\mathrm{MAPbI}}_{3}\left(\mathrm{Cl}\right)$ film is more stable than ${\mathrm{MAPbI}}_{3}$ under atmosphere exposure. In order to hinder $O_{2}^{-}$ ion formation and stop the degradation of the material, authors propose a treatment with iodine salts. They, indeed, substitute oxygen molecules in the process of recombination with iodine vacancies, suppressing the formation of the superoxide species and reducing the number of non-radiative trap sites. Moreover, iodine salts treatment does not modify the perovskite structure, leading the process of oxygen diffusion unchanged.

Recently, fully inorganic perovskites have been discovered to be excellent alternatives to their hybrid counterparts since, by substituting the organic cation with a cesium ion into the perovskite, the structure gains major stability and suffers less long term degradation phenomena.

Nevertheless, to date, there are very few reports in literature which examine in depth the effects of the interaction between the inorganic perovskite and the ambient air (such as oxygen and water molecules, alone or combined), and provide details about the morphology of the film, the photoluminescence and electrical properties of the sample before and after the exposure to the ambient gas. As a consequence, lots of questions about the topic remain unsolved.

Photoluminescence measurements of ${\mathrm{CsPbBr}}_{3}$ perovskite nanocrystals in controlled oxygen atmosphere reveal strong quenching, which is in contrast to the photobrightening effect observed in both ${\mathrm{MAPbX}}_{3}$ and ${\mathrm{CsPbX}}_{3}$ perovskite films and single crystals [59,65,70,72].

In order to investigate the optical response to environmental gases of CsPbBr${}_{3}$ perovskite nanocrystals, the effects of the atmosphere in the exciton recombination processes and the nature of trap states, Lorenzon et al. [79] conducted PL measurements combined with spectro-electrochemical analysis.

The application of electrochemical potentials induces the variation of the Fermi level position, which modulates the emission intensity by altering the occupancy of defect states without degrading the nanocrystals. It has been observed that when a negative reductive potential is applied, the PL emission is strongly quenched, whereas upon the application of a positive oxidative potential the emission slightly increases. In particular the reductive potential leads to the raising of the Fermi level, and the emission mechanism is mostly influenced by trapping of photogenerated holes, whereas electron trapping has a negligible role in nonradiative PL quenching; on the contrary, an oxidative potential corresponds to the lowering of the Fermi level leading to the suppression of hole trapping in defect states. The difference between the PL emission, upon negative and positive electrochemical potential, and the relatively high PL quantum efficiency in unperturbed conditions suggest that the number of active intragap traps is very small and, as a consequence, the oxygen molecules directly interact with the photogenerated electrons in the conduction band without the mediation of the structural defects.

This behavior confirms that the interaction mechanism between CsPbBr${}_{3}$ perovskite NCs and molecular oxygen is clearly different from the process leading to the photobrightening observed in perovskites films and single crystals when exposed to oxygen, which is ascribed to the passivation of defect states by adsorption of O${}_{2}$ molecules on the sample surface.

The ambient effects on the degradation processes of fully inorganic perovskite nanocrystals (NCs) when illuminated with UV radiation have been deeply investigated also in [62,63].

**Figure 9:**
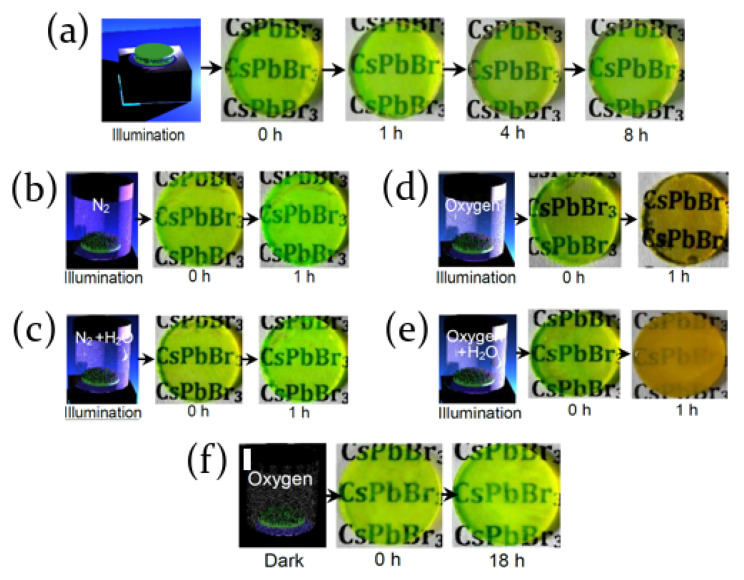
Optical images of the ${\mathrm{CsPbBr}}_{3}$ film (**a**) sandwiched between two quartz coverslips as a function of the illumination time (175 $\mathrm{mW}/{\mathrm{cm}}^{2}$, RH 60%). Optical images of the ${\mathrm{CsPbBr}}_{3}$ film exposed to (**b**) pure $N_{2}$ in an illuminated environment, (**c**) $N_{2}$ + 0.5 μL $H_{2}O$ in an illuminated environment, (**d**) oxygen in an illuminated environment, (**e**) oxygen + 0.5 μL $H_{2}O$ in an illuminated environment and (**f**) oxygen in a dark environment. Adapted with permission from [62]. Copyright © 2017, American Chemical Society.

As an example, Huang et al. [62] performed several experiments on ${\mathrm{CsPbBr}}_{3}$ NCs both in toluene solution and as thin films, testing the role of illumination power density, moisture and oxygen, and concluding that the NCs surface decomposition is due to a synergistic combination of the three elements (Figure 9a–f). It has been demonstrated indeed that in presence of light, humidity sustains the degradation process (Figure 9e), whereas in dark conditions the effect of oxygen and RH is negligible, as evidenced in Figure 9f.

Indeed, oxygen and moisture can reach perovskite surface and the NCs interfaces thanks to their hydrophilic properties, so that ${\mathrm{CsPbBr}}_{3}$ hydrates species can be formed. As an effect of illumination, surface bonding ligands are removed through photons absorption. The energetic barrier among crystals becomes weaker, and thus less stable, and NCs tend to aggregate.

Oxygen molecules can erode the NCs, while moisture presence enhances ion migration leading to the formation of PbO and ${\mathrm{PbCO}}_{3}$ species on the perovskite surface. Surface modification contribute to the generation of trap states, resulting in decreased PL emission [62,63].

### 2.3. Gas Atmosphere Effects ( $NH_{3}$, $NO_{2}$, VOC, $O_{3}$, $H_{2}$)

The evidence of possible reversible modulation of the active properties of perovskite thin films stimulated experiments aiming to exploit this effect, in order to detect gas analytes in the sample environment, with particular interest toward pollutants and gases hazardous for human health and environment.

In this section we will resume the main results on the interaction between perovskites and several gas species.

#### 2.3.1. Ammonia

The detection of ${\mathrm{NH}}_{3}$ molecules is of high importance since they represent one of the most harmful pollutants; checking the level of ammonia in the atmosphere thus represents the first step to avoid problems on human health. In this regard, metal halide perovskites turned out to be fundamental since their high sensitivity to ammonia presence has been demonstrated both for hybrid and fully inorganic active materials, becoming candidates for the development of a sensing device.

**Figure 10:**
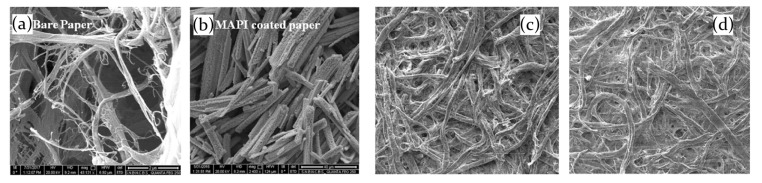
Field emission scanning electron microscope (FESEM) images of (**a**) bare paper and (**b**) ${\mathrm{MAPbI}}_{3}$-coated paper: perovskite exploits the fibril structure of the paper substrate to grow with a nanorod-like structure. FESEM images of (**c**) ${\mathrm{PbI}}_{2}$ and (**d**) ${\mathrm{MAPbI}}_{3}$ after ${\mathrm{NH}}_{3}$ exposure: the morphology of the ${\mathrm{MAPbI}}_{3}$ film after interacting with ammonia looks like ${\mathrm{PbI}}_{2}$ film more than the pristine perovskite film. Adapted with permission from [80], under the terms of the Creative Commons Attribution 4.0 International License. Copyright © 2018, Springer Nature.

Maity and his coworkers deeply investigated the interaction of hybrid lead I-based perovskite with ${\mathrm{NH}}_{3}$ molecules, concluding than the main effect is the decomposition of the active material into ${\mathrm{PbI}}_{2}$ [80,81]. Interestingly, scanning electron microscopy (SEM) images of ${\mathrm{MAPbI}}_{3}$ films deposited on a paper substrate prove that after the interaction with the ammonia the perovskite morphology is more similar to the ${\mathrm{PbI}}_{2}$ than to the pristine ${\mathrm{MAPbI}}_{3}$, as evidenced in Figure 10a–d [80]. Moreover, also the visual aspect of the film evidently suffers from the interaction, since a color change is observed from perovskite natural dark brown/black to a pale yellow, whose shade is influenced from ${\mathrm{NH}}_{3}$ gas concentration.

In this regard, gas concentration resulted fundamental for the sensing properties of the sample. Considering the color change of the sample, the response time of the active mean, that is the time needed to reach the 90% of the maximum of the response, exponentially decreases with higher gas concentrations. At low ${\mathrm{NH}}_{3}$ concentration (10 ppm) the film changes its color with a response time of about 12 s, and reaches 3 s for 30 ppm of gas content. Moreover, it has been demonstrated that for low ${\mathrm{NH}}_{3}$ concentration (10 ppm) the transformation of ${\mathrm{MAPbI}}_{3}$ perovskite in ${\mathrm{PbI}}_{2}$ molecules is reversible upon removal of the gas source, whereas it becomes irreversible for higher concentration (30 ppm), leading to a detrimental perovskite disintegration, thereby imposing an upper limit to the sensor application [80].

However, the effects of the interaction of hybrid lead halide perovskite with ammonia are not totally clear yet, since several authors support the idea that the exposition to ${\mathrm{NH}}_{3}$ induces the formation of new unknown non-luminescent phases, not attributable to ${\mathrm{PbI}}_{2}$ molecules [82,83,84,85].

Bao et al. [84], for example, found that ${\mathrm{MAPbI}}_{3}$ perovskite films interacting with ${\mathrm{NH}}_{3}$ transform in a new unknown compound, named (${\mathrm{MAPbI}}_{3}\;+\;{\mathrm{NH}}_{3}$), which visually appears as a color change from typical perovskite dark brown to a light yellow. Interestingly, they found that, even if the phase transition becomes irreversible after only 50 s, the permanent modification of the film appearance is not accompanied by a modification of the electrical behavior of the sample. The new (${\mathrm{MAPbI}}_{3}\;+\;{\mathrm{NH}}_{3}$) compound seems indeed to respond to ammonia presence identically to pristine perovskite, changing its resistance as a function of the on/off ${\mathrm{NH}}_{3}$ cycles.

Structural modifications of perovskite samples in presence of ammonia have also been explored by Singh and his coworkers [82], who showed that both bare and PMMA-covered ${\mathrm{MAPbBr}}_{3}$ quantum dots (QDs) lost their photoluminescence emission when interacting with ${\mathrm{NH}}_{3}$ molecules. After exposing ${\mathrm{MAPbBr}}_{3}$ QDs to ammonia, ${\mathrm{NH}}_{4}^{+}$ cations are thought to replace methylammonium cations (${\mathrm{CH}}_{3}{\mathrm{NH}}_{3}^{+}$) in perovskite, leading to the formation of ${\mathrm{NH}}_{4}{\mathrm{PbBr}}_{3}$ species. However, on the removal of the ${\mathrm{NH}}_{3}$ source, the reaction does not turn back alone and the injection of ${\mathrm{CH}}_{3}{\mathrm{NH}}_{2}$ molecules is needed to restore pristine perovskite structure and its PL emission.

Similarly, Ruan et al. [83] found that metal halide perovskite crystals and films interacting with ammonia create a new composite, starting from the oxidation process of ${\mathrm{NH}}_{3}$ to ${\mathrm{NH}}_{4}^{+}$. In this case, however, reduced methylammonium ion (${\mathrm{MA}}^{+}$ to MA) is thought to remain inside the perovskite crystal structure, forming a weakly coordinated complex (${\mathrm{NH}}_{4}{\mathrm{PbX}}_{3}\cdot \mathrm{MA}$); the weak bound with MA can be thermally broken, restoring pristine perovskite crystal structure. Interestingly, experimental XRD measurements evidenced that this is only partially true, since the chemical composition of the perovskite influences its stability to external stimuli. Only ${\mathrm{MAPbCl}}_{3}$ single crystals have been found to be completely restored when the ${\mathrm{NH}}_{3}$ source is removed, whereas ${\mathrm{MAPbBr}}_{3}$ and ${\mathrm{MAPbI}}_{3}$ showed traces of the ${\mathrm{NH}}_{4}{\mathrm{PbX}}_{3}\cdot \mathrm{MA}$ complex in their XRD patterns after ammonia treatment.

**Figure 11:**
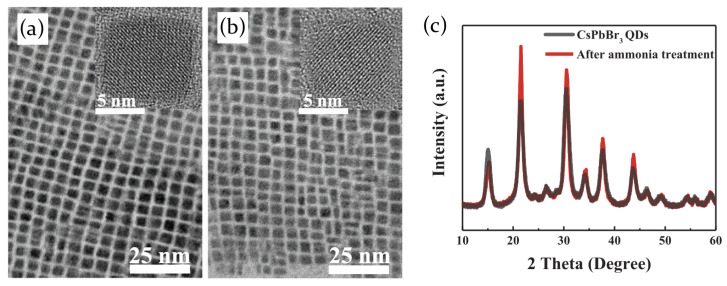
TEM images of ${\mathrm{CsPbBr}}_{3}$ QDs (**a**) before and (**b**) after ammonia gas exposure. (**c**) XRD pattern of ${\mathrm{CsPbBr}}_{3}$ QDs before and after ${\mathrm{NH}}_{3}$ treatment. Adapted with permission from [86]. Copyright © 2020, WILEY-VCH Verlag GmbH and Co. KGaA, Weinheim.

Contrary to hybrid perovskites, recently it has been demonstrated that fully inorganic ${\mathrm{CsPbBr}}_{3}$ perovskite QDs do not suffer from structural modification induced by the interaction with ammonia [86]. XRD patterns of perovskite before and after ${\mathrm{NH}}_{3}$ exposure have indeed been shown to be quite similar (Figure 11c) and measurements on morphology additionally confirm that the size (≃10 nm) and the cubic phase of the quantum dots remain unaltered (Figure 11a,b). As a consequence, the interaction between inorganic perovskite and ammonia is physical rather than chemical, with ${\mathrm{NH}}_{3}$ assuming the role of passivating agent for surface defects through the interaction with Pb ions. The reduction of trap states density is confirmed by TA measurements and leads to the enhancement of the sample PL emission in presence in ammonia.

#### 2.3.2. Nitrogen Dioxide

**Figure 12:**
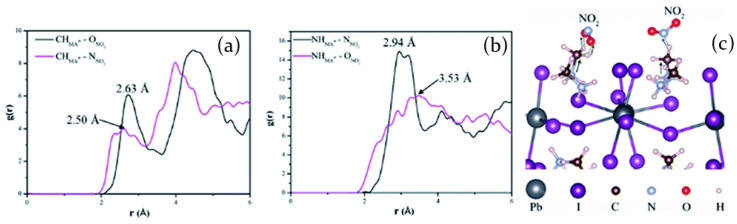
Radial distribution functions (RDF) of adsorbed ${\mathrm{NO}}_{2}$ molecules on the (110) face of ${\mathrm{MAPbI}}_{3}$, including both hydrogen bond interactions with (**a**) $-{\mathrm{CH}}_{3}$ (peaks 2.50 Åand 2.63 Å) and (**b**) $-{\mathrm{NH}}_{3}$ (peaks at 2.94 Åand 3.53 Å). (**c**) Schematic illustration of the electron transfer process from perovskite to the ${\mathrm{NO}}_{2}$ molecules via the hydrogen bond channel. Adapted with permission from [87] under the terms of the Creative Commons Attribution 3.0 Unported license. Copyright © 2018, Royal Society of Chemistry.

${\mathrm{MAPbI}}_{3}$ perovskite is demonstrated to be highly sensitive also to ${\mathrm{NO}}_{2}$[87], one of the most common harmful chemicals for human health. In presence of ${\mathrm{NO}}_{2}$, both at atmospheric pressure and high pressure, the ${\mathrm{MAPbI}}_{3}$ films increase their conductivity. Then, the conductivity decreases back to the initial value when the films are exposed to an inert gas. On the basis of computational simulations and experimental Fourier transform infrared spectroscopy (FTIR) results, a simple model is proposed to describe the interaction between the gas and the surface atoms, ascribed to electron transfer from perovskite to ${\mathrm{NO}}_{2}$ molecules (Figure 12a–c). Nitrogen dioxide molecules, in their approach to perovskite surface, are thought to interact with the methyl group -${\mathrm{CH}}_{3}$ rather than the -${\mathrm{NH}}_{3}$ one.

This effect is confirmed by computational analysis in which Radial Distribution Functions (RDF) of ${\mathrm{NO}}_{2}$ molecules adsorbed on the (110) face of ${\mathrm{MAPbI}}_{3}$ have been calculated and reported in Figure 12a,b. In particular, Figure 12a evidences the RDFs ascribed to the bond between ${\mathrm{NO}}_{2}$ and -${\mathrm{CH}}_{3}$ group of ${\mathrm{MA}}^{+}$ ion through the O atom (black line) and the N atom (red line), whereas in Figure 12b the RDFs between N and O atoms of ${\mathrm{NO}}_{2}$ and the -${\mathrm{NH}}_{3}$ group of ${\mathrm{MA}}^{+}$ are reported in black and red, respectively. A better overlap of the RDFs first peaks at 2.50 Å and 2.63 Å (Figure 12a) obtained for the interaction between ${\mathrm{NO}}_{2}$ molecules and -${\mathrm{CH}}_{3}$ group reveals a stronger hydrogen bond than that obtained from the interaction between ${\mathrm{NO}}_{2}$ and -${\mathrm{NH}}_{3}$ group, evidenced by peaks at 2.94 Å and 3.53 Å. Once the hydrogen bond is formed, a charge transfer can occur. Starting from the perovskite Pb-I skeleton, which is known to represents the major channel of charge transport [88], some electrons are transferred to ${\mathrm{MA}}^{+}$ ions and then out of perovskite, leaving the ${\mathrm{MAPbI}}_{3}$ crystal structure with a lack of negative charge. The hole concentration increase induces a resistance decrease, and subsequently, a current enhancement. As a consequence, the higher the ${\mathrm{NO}}_{2}$ pressure is, the more efficient the electron transfer becomes, resulting in an increase of current in the device.

#### 2.3.3. Volatile Organic Compound

Volatile organic compound (VOC) gases contribute to ambient air pollution [89], in particular in indoor space, leading to health problems such as respiratory diseases [90], eye irritation [91], skin allergy [92], fatigue [93], and central nervous system disorder [94]. For this reason finding VOC sensors with high sensitivity and fast response, able to operate at room temperature, would be very important [7].

Nur’aini et al. [7] have shown that ${\mathrm{MAPbI}}_{3}$ perovskite thin films are very sensitive to the presence of VOC gases in the atmosphere and then are good active materials for sensors operating at room temperature, with good reversibility and repeatability. In particular, electrical properties of perovskite films are tested by applying a voltage bias to the interdigitated electrode and measuring the current-time response of the film. Samples are tested toward a range of typical organic VOCs, both polar gases, such as ethanol, acetone, isopropanol, acetonitrile and methanol, and non-polar gases, such as toluene, benzene, chloroform, and hexane, always obtaining an increase of the current in presence of gas and a subsequent current decrease when the atmosphere is switched back to inert gas.

A sensing mechanism based on charge trap state passivation is proposed. ${\mathrm{MAPbI}}_{3}$ is well known to contain iodine vacancies, which could be located in MA-I and Pb–I layers [95]. As MA-I layer is a termination layer of ${\mathrm{MAPbI}}_{3}$, most of iodine vacancies are located on the surface, interfacial sites, and grain boundaries. During the film deposition, the solvent evaporation leads to the formation of a high density of crystal defects which act as trap states. In pristine film, electrons can fall from the conduction band into the iodine vacancies trap states, and as a consequence, the film is characterized by low conductivity. In presence of an ambient gas, the gas molecules passivate the iodine vacancies and the trapped electrons can be restored into the conduction band, determining an increase of the conductivity. If an inert gas is then injected to recover the perovskite, the absorbed inert gas molecules expel the VOC gas ones and recreate vacancies, re-establishing the crystal defects of the perovskite film and reducing its conductivity. This sensing mechanism is supported also by the photoluminescence measurements, which highlight a PL increase after the exposition to the gas and a return to lower PL intensity on the removal of the gas.

**Figure 13:**
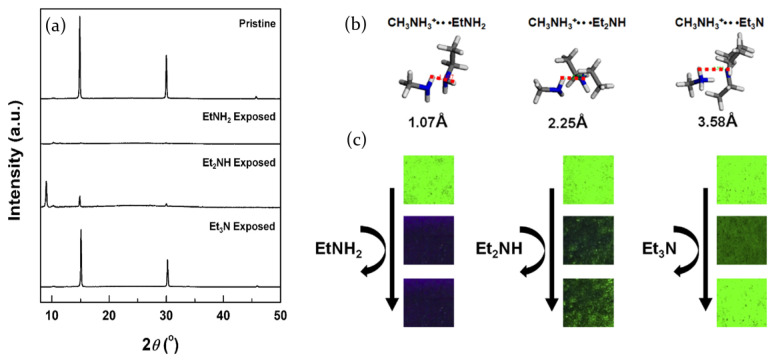
(**a**) X-ray diffraction (XRD) patterns of ${\mathrm{CH}}_{3}{\mathrm{NH}}_{3}{\mathrm{PbBr}}_{3}$ films before amine exposure (pristine), after exposure to ${\mathrm{EtNH}}_{2}$, ${\mathrm{Et}}_{2}\mathrm{NH}$ and ${\mathrm{Et}}_{3}N$. (**b**) Interaction distances between ${\mathrm{MA}}^{+}$ of perovskite and aliphatic amines obtained through DFT calculation. (**c**) Photographs of ${\mathrm{MAPbBr}}_{3}$ perovskite films recorded under 365 nm UV light after exposure to ${\mathrm{EtNH}}_{2}$, ${\mathrm{Et}}_{2}\mathrm{NH}$ and ${\mathrm{Et}}_{3}N$. Reprinted from [96]. Copyright © 2017, with permission from Elsevier.

The effects of volatile organic compounds (VOCs) on the photoluminescence properties of lead bromide perovskite have also been investigated by Kim et al. [96], who focused on the role of aliphatic amines. ${\mathrm{MAPbBr}}_{3}$ thin films have been exposed to three different amine vapors with different aliphatic chains: monoethylamine (${\mathrm{EtNH}}_{2}$), diethylamine (${\mathrm{Et}}_{2}\mathrm{NH}$) and trimethylamine (${\mathrm{Et}}_{3}N$). In response to the amine vapor exposure the perovskite films suffered photoluminescence quenching. Anyway ${\mathrm{MAPbBr}}_{3}$ showed full recovery only after exposure to ${\mathrm{Et}}_{3}N$, whereas the ${\mathrm{Et}}_{2}\mathrm{NH}$ exposed film was characterized by a slower and only partial PL recovery, and ${\mathrm{EtNH}}_{2}$ vapor determined an irreversible quenching of the photoluminescence. XRD measurements evidenced that fluorescence quenching originates from the structural changes in the perovskite films (Figure 13a) caused by the interaction with the aliphatic amines. In particular, the films exposed to ${\mathrm{EtNH}}_{2}$ and ${\mathrm{Et}}_{2}\mathrm{NH}$ clearly show full and partial degradation of the crystal structure, while no structural changes are observed in the film exposed to ${\mathrm{Et}}_{3}N$, thereby explaining the different behavior in terms of reversibility.

The alteration of the perovskite crystal structure is due to the intercalation of polar molecules into the lattice, which breaks the hydrogen bonds of the perovskite and forms new ones with the halides. Then, in order to investigate the origin of the observed reversible/irreversible changes in emission signals, density functional theory (DFT) and molecular electrostatic potential (MEP) calculations have been performed. The hydrogen bonding distances between perovskite MA${}^{+}$ ions and aliphatic amines are calculated to be 1.07 Å, 2.25 Åand 3.58 Åfor ${\mathrm{EtNH}}_{2}$, ${\mathrm{Et}}_{2}\mathrm{NH}$ and ${\mathrm{Et}}_{3}N$, respectively (Figure 13b); it suggests that, due to the longer hydrogen bonding distance, the interaction strength between ${\mathrm{Et}}_{3}N$ and ${\mathrm{MA}}^{+}$ is lower, allowing reversible photoluminescence quenching. This behavior is confirmed by the MEP calculations that indicate a significant decrease of negative potential of nitrogen atom in ${\mathrm{Et}}_{3}N$ and confirm its relatively lower hydrogen bonding strength with perovskite. Vice versa the shorter distance, and then the higher interaction strength, between ${\mathrm{EtNH}}_{2}$ and ${\mathrm{MA}}^{+}$ results in irreversibility.

The possibility to develop metal halide gas sensors has also been investivated for fully inorganic perovskites. Porous layers of ${\mathrm{CsPbBr}}_{3}$ have been indeed demonstrated to be highly sensitive to the presence of volatile organic compounds, showing a modulation of electrical properties under visible light excitation. In particular, the samples have been analyzed under exposure to acetone and ethanol, and also to oxygen, showing a clear enhancement of the photocurrent in presence of the target gas and a decrease when, subsequently, is exposed to inert atmosphere. Moreover, the choice of using different gas sources allowed to demonstrate that the perovskite is able to respond with an equal current change to both oxidizing and reducing gas, due to the perovskite ambipolar charge transport due to the similarity of electron effective mass and hole effective mass [9,49,97].

Lead bromide perovskites in pristine conditions—in an inert atmosphere—are characterized by a high density of bromide vacancies, which act as trap states for photoexcited charges. As a consequence, the conductivity of the sample results unavoidably low. On the contrary, when the sample is exposed to an external gas source, such as oxygen, ethanol or acetone, the gas molecules act as trap fillers for bromide vacancies. Decreasing the density of trap states means a higher number of photoexcited charges available for electrical transport, which translates into a photocurrent increase. The overall process is reversible, so when the chamber is evacuated, gas molecules desorb from the perovskite surface, restoring the initial trap states density condition.

#### 2.3.4. Ozone

A gas-sensing mechanism based on the surface trap passivation has also been hypothesized to explain the interaction between ozone molecules and the surface of lead mixed halide perovskite ${\mathrm{MAPbI}}_{3-x}{\mathrm{Cl}}_{x}$ thin films, resulting in a decrease of the electrical resistance of the sample [98]. The gas molecules, adsorbed within the perovskite lattice close to the surface, passivate the traps (unpaired ${\mathrm{Pb}}^{2+}$ ions), and as a result, the sensing films resistance is reduced. In detail, the increase of the current through the perovskite is due to the electron transfer from the $O_{3}$ to the ${\mathrm{Pb}}^{2+}$ cations. Consequently, the excess of positive charges is neutralized, the surface recombination rate in the film is modulated and the surface trap passivation leads to the enhancement of the conductivity, due to a lower hole-electron carrier trapping. Such a change in the conductivity depends on the amount of ozone concentration molecules adsorbed onto the surface and consequently on the surface morphology of the film (grain size, porosity, roughness). When the sample chamber is evacuated, the ozone molecules are quickly desorbed, and thus the perovskite film restores its initial electrical conductivity [99] in very short response times.

**Figure 14:**
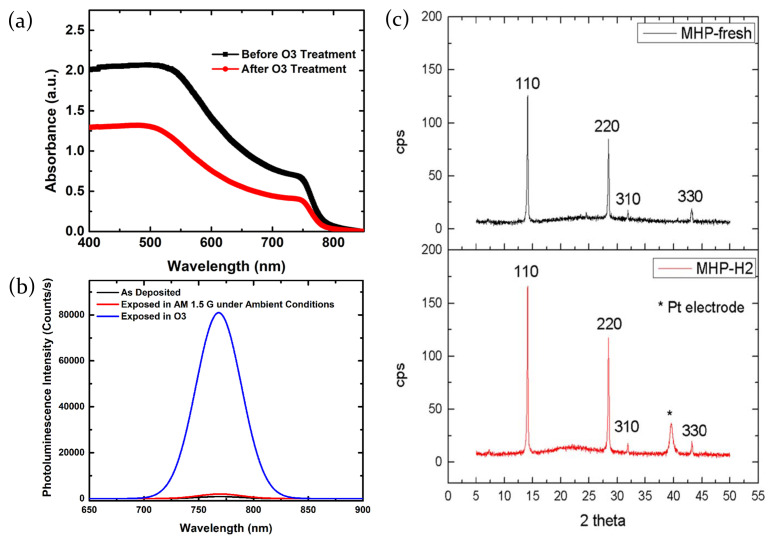
(**a**) UV–Vis absorption measurements before and after $O_{3}$ exposure for ${\mathrm{MAPbI}}_{3-x}{\mathrm{Cl}}_{x}$ perovskite films. (**b**) PL spectra of the pristine (black line), Solar A.M. 1.5G exposed (red line) and $O_{3}$ exposed (blue line) ${\mathrm{MAPbI}}_{3-x}{\mathrm{Cl}}_{x}$ perovskite films. Adapted with permission from [98]. Copyright © 2017, American Chemical Society. https://pubs.acs.org/doi/10.1021/acssensors.7b00761. Further permission should be directed to the ACS. (**c**) XRD patterns of ${\mathrm{MAPbI}}_{3-x}{\mathrm{Cl}}_{x}$ films before and after exposure to 100 ppm hydrogen gas, which demonstrate that the perovskite does not change crystal structure after interacting with $H_{2}$. The only different feature noticeable in $H_{2}$-exposed perovskite film relates to the Pt electrode; it is absent in the pristine sample spectra, since the XRD of the non-exposed sample was done on a glass substrate without electrodes. Adapted with permission from [8] under the terms of the Creative Commons Attribution 3.0 license. Copyright © 2020, IOP Science.

The hypothesized sensing process is confirmed by the photoluminescence measurements which demonstrate that, after the exposure of the film to ozone, PL intensity increases significantly. Indeed, due to the $O_{3}$ passivation of the surface traps in the perovskite films, the rate of the non-radiative recombination [99] decreases. Additionally, the absence of shift of the peak PL wavelength indicates the absence of substantial crystal phase change during the ozone exposure (Figure 14b).

Moreover, it has been verified that a prolonged exposure to ozone at high gas concentration can induce detrimental effect in the perovskite. As evidenced in Figure 14a, the exposure of the ${\mathrm{MAPbI}}_{3-x}{\mathrm{Cl}}_{x}$ thin films to 2500 ppb ozone concentration leads to a uniform drop of the UV-Vis absorption spectra intensity after about 60 min. However, such high levels of ozone exposure are quite drastic, so in standard conditions (gas concentration lower than 75 ppb, which is the safety limit imposed by International Agencies) [100] the sensing process can be considered fully reversible.

#### 2.3.5. Hydrogen

Lead mixed halide perovskite ${\mathrm{MAPbI}}_{3-x}{\mathrm{Cl}}_{x}$ thin films are sensitive also to hydrogen ($H_{2}$) [8]. Hydrogen is a reducing gas, and since the ${\mathrm{MAPbI}}_{3-x}{\mathrm{Cl}}_{x}$ is a p-type semiconductor, the adsorption of the $H_{2}$ molecules causes the lowering of the current. The interaction between the perovskite and the gas is typically physical rather than chemical: the $H_{2}$ molecules, adsorbed through the porous of the perovskite film, bond loosely close to the surface and leave the film after the removal of the gas, without inducing any structure modification. This is confirmed by the XRD patterns of the sample taken before and after the exposure to $H_{2}$. They indeed show the same peaks without substantial differences in intensity (Figure 14c).

Under $H_{2}$ exposure the film resistance increases because, being the hydrogen a reducing gas, it releases electrons that recombine with the holes (majority charge carriers in the p-type semiconductor) resulting in the lowering of the current through the film. When hydrogen is desorbed the resistance of the material returns to the its initial value.

## 3. Sensing Application of Perovskites

The reversible response to the environment makes the perovskite-based films attractive sensing elements in the detection of harmful gases. It has been observed that sometimes the exposition to the surrounding atmosphere does not induce any phase transformation, indicating a non-chemical interaction between the target gas and the perovskite, and it has been demonstrated that in these cases the surface traps play an important role in determining the gas sensing mechanism both in the optical and the electrical response.

The conductivity of the perovskite layers is greatly influenced by the environmental gases and the electrical response to both oxidizing and reducing gases, as already evidenced, is symmetrical. This behavior indicates a sensing mechanism in perovskites different from that of semiconductors typically employed in MOS technology. Indeed, because of the similarity of electron and hole effective masses in halide perovskites, the charge transport is bipolar leading to analogous variation of photocurrent with increasing concentrations of oxidizing or reducing gases. For this reason these materials are able to operate as sensors for both the types of analytes.

To date the most diffused approach reported in literature is the realization of hybrid lead halide perovskites gas sensors based on the modulation of the electrical properties, induced by the atmosphere chemical composition variation [81,84].

On the contrary, the development of lead halide perovskites optical sensors, exploiting the reversible photoluminescence alterations due to the interaction with the analyte molecules, is much less developed, with limited examples in literature.

### 3.1. Resistive Sensors

**Figure 15:**
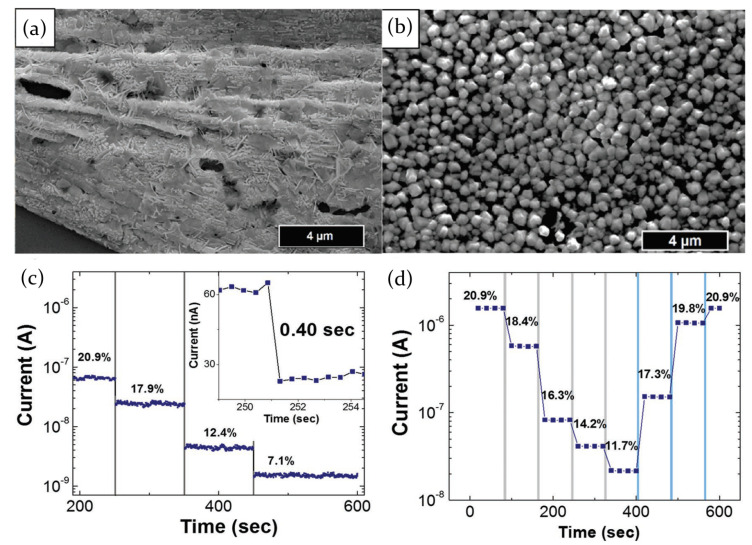
SEM images of (**a**) 1S sample and (**b**) 2S sample of ${\mathrm{MAPbI}}_{3}$ polycrystalline films, showing the different morphologies coming from the deposition technique used. (**c**) Current change at decreasing oxygen levels. Inset: magnification of the current plot showing the response time of the device. (**d**) Current plot highlighting the reversibility of the sensor for decreasing (from 20.9% to 11.7%) and then increasing (from 11.7% to 20.9%) $O_{2}$ gas concentration. Adapted with permission from [66]. Copyright © 2017, WILEY-VCH Verlag GmbH and Co. KGaA, Weinheim.

The first experimental demonstration of the use of perovskite-based device as a resistive sensor has been provided by Stoeckel et al. [66], who exploited the interaction of ${\mathrm{MAPbI}}_{3}$ polycrystalline films with oxygen. A more-than-three order of magnitude increase in current has been measured changing the atmosphere from pure $N_{2}$ to $O_{2}$. Oxygen molecules diffuse inside the perovskite crystal structure and reversibly fill iodine vacancies, which act as sensing sites. This determines a reduced carrier trapping resulting in a conductivity, and thus current, increase. The trap healing mechanism does not involve the creation of a strong covalent bond between $O_{2}$ molecules and perovskite, but it is just a physical interaction guided by the gas concentration in the chamber. It then leads to a fast response of the device (400 ms) and a full reversibility of the process (Figure 15c,d).

The analysis, moreover, revealed that the morphology, depending on the deposition technique, strongly influences the response to an external stimulus. Two samples were prepared through solution-based deposition methods, in one (1S) or two steps (2S). In the 2S approach, a ${\mathrm{PbI}}_{2}$ solution was spin coated on a $\mathrm{Si}/{\mathrm{SiO}}_{2}$ substrate, followed by the deposition of a MAI solution on the previously prepared ${\mathrm{PbI}}_{2}$ film; on the other hand, the 1S technique directly involved the deposition of an equimolar mixture of the two precursors. The sample 2S is characterized by a more uniform coverage (Figure 15b), with very small crystals (about 500 nm in length and 400 nm in thickness), which maximizes the diffusion of oxygen molecules in the film, and as a consequence, the sensing response. In a 95% $O_{2}$ atmosphere almost all traps in the film result to be filled. On the contrary, fibril-like structure obtained in the 1S sample (Figure 15a) does not allow oxygen to diffuse properly in the sample, resulting in a less efficient sensing response.

Furthermore, the device has also been tested with other gases in order to investigate the selectivity of the response, that is the ability to respond only to a single gas source, that represents one of the main properties of a correctly working sensor. ${\mathrm{SO}}_{2}$ and ${\mathrm{NH}}_{3}$ have been shown to induce a current change much lower than that induced by oxygen molecules; the presence of moisture, instead, leads to the formation of electrically insulating species, detrimental for the sensor.

**Figure 16:**
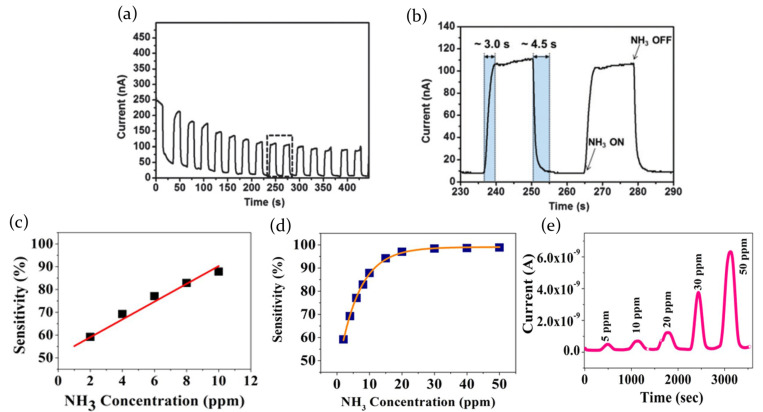
(**a**) I–t curve for a ${\mathrm{MAPbI}}_{3}$ perovskite film for ${\mathrm{NH}}_{3}$ on/off cycles; the current plot shows an initial overall gradual decrease due to the phase transformation, then settling at a constant level. (**b**) Expanded view of the on and off switches of the gas, with the corresponding response and recovery times (blue shade). Used with permission from the Royal Society of Chemistry [84]. Copyright © 2015. Permission conveyed through Copyright Clearance Center, Inc. (**c**) Linear dependence of the sensitivity as a function of ${\mathrm{NH}}_{3}$ concentration in a 1–10 ppm range for a ${\mathrm{MAPbI}}_{3}$ film deposited on a paper substrate; (**d**) sensitivity as a function of higher values, ammonia concentration 1–50 ppm ; (**e**) current modulation resulting from ${\mathrm{NH}}_{3}$ on/off switches at increasing gas concentration. Adapted with permission from [81] under the terms of the Creative Commons Attribution 4.0 International License. Copyright © 2019, Springer Nature.

The process of physical adsorption of atmosphere molecules in ${\mathrm{MAPbI}}_{3}$ perovskite crystal lattice has been also verified by Bao et al. [84] in presence of ${\mathrm{NH}}_{3}$ as target gas. The exposition to the chosen gas has been found to induce an immediate current increase, with a reversible behavior in case of gas evacuation. Besides, authors interestingly noticed how this reversible current modulation was accompanied by an irreversible phase transformation, showing a resistance higher than the pristine perovskite, but similar sensing ability. As a results, switching on and off the ${\mathrm{NH}}_{3}$ flow, current plot is characterized by an overall gradual decrease in the first few minutes of the measurement. Once the perovskite is completely transformed in the new phase, the current sets up on a constant level, following the ${\mathrm{NH}}_{3}$ on/off cycles with a corresponding current increase/decrease, with a response time and a recovery time, respectively, of 3 s and 4.5 s (Figure 16a,b).

Current variation after the interaction of ${\mathrm{MAPbI}}_{3}$ perovskite with ${\mathrm{NH}}_{3}$ gas has also been investigated by Maity et al. [81], and the electrical response has been related to the formation of ${\mathrm{PbI}}_{2}$ on the surface of the active layer. The perovskite film, deposited on a paper substrate, consists of nano and microrods, with an average length of 30 μm and a diameter ranging from 0.7 to 1.6 μm. The exposure to ${\mathrm{NH}}_{3}$ leads to perovskite transformation into ${\mathrm{PbI}}_{2}$ that has a higher conductivity, thereby causing a current increase (Figure 16e). However, the gas concentration plays an important role in the process, since only for low values (<10 ppm) the reaction mechanism is fully reversible. At increasing gas pressures, the transformation of perovskite into ${\mathrm{PbI}}_{2}$ goes on, and allows reversible current switches only for short time exposure. The process then becomes totally irreversible for ${\mathrm{NH}}_{3}$ concentration higher than 50 ppm.

The sensitivity S of the sample (defined as the ratio $\Delta R/R_{0}$, where ΔR = $R_{0}-R_{g}$ is the variation of resistance with $R_{g}$ the resistance when the sensor is exposed to the gas (NH${}_{3}$) and $R_{0}$ the resistance in inert atmosphere) reflects this behavior. It indeed linearly depends on the gas concentration for low values (<10 ppm), and saturates after reaching 30 ppm, as shown in Figure 16c,d. However, the device has shown to be able to detect low gas concentration down to 1 ppm with a 55% sensitivity and reaches sensitivity of almost 90% at 10 ppm. The response and recovery times have also been investigated, both being of about 120–130 s at a ${\mathrm{NH}}_{3}$ concentration of 10 ppm.

This experiment investigated also the selectivity of the response by testing the device with other volatile compounds, such as ethanol, methanol, TCE (trichloro ethelyne) and IPA (2 propanol). All of them have shown to induce a small negative sensitivity response, which means that the resistance of the film increases when exposed to those gases. Only acetone induced a current increase, even if the response, compared to ${\mathrm{NH}}_{3}$ one, is negligible.

**Figure 17:**
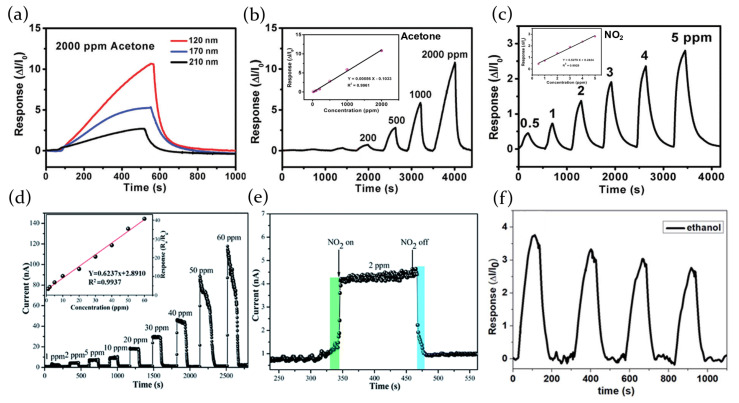
(**a**) Response of the SCN-organometal halide perovskites-based sensor with a 120, 170 or 210 nm-thick sensing layer when exposed to 2000 ppm of acetone. (**b**) Current response of the 120 nm thick perovskite layer to increasing acetone concentrations. Inset: linear dependence of the current response of the sensor to the gas concentration, showing an average sensitivity of $5.6\cdot 10^{-3}\;{\mathrm{ppm}}^{-1}$. (**c**) Current response of the 120 nm thick perovskite layer to ${\mathrm{NO}}_{2}$ concentrations ranging from 500 ppb to 5 ppm. Inset: linear increase of the device response to the gas concentration, resulting in an average sensitivity of $5.3\cdot 10^{-1}\;{\mathrm{ppm}}^{-1}$. Used with the permission of the Royal Society of Chemistry, from [49]. Copyright © 2017. Permission conveyed through Copyright Clearance Center, Inc. (**d**) Response curves of the ${\mathrm{MAPbI}}_{3}$ film sensor at different ${\mathrm{NO}}_{2}$ concentrations (1–60 ppm). Inset: linear dependence of the resistive response to the gas concentration, resulting in an average sensibility of 0.62 ppm${}^{-1}$. (**e**) Expanded view of the on and off switches of the gas, with the corresponding response and recovery processes (green and blue shades). Adapted with permission from [87] under the terms of the Creative Commons Attribution 3.0 Unported license. Copyright © 2018, Royal Society of Chemistry. (**f**) Response–time graph of ${\mathrm{MAPbI}}_{3}$ film when exposed to on/off switches of ethanol gas. Adapted with permission from [7] under a Creative Commons Attribution-NonCommercial 3.0 Unported license. Copyright © 2020, Royal Society of Chemistry.

The quick room-temperature reversible behavior of ${\mathrm{MAPbI}}_{3}$ films when exposed to nitrogen dioxide indicates their potential application as ${\mathrm{NO}}_{2}$ sensors, showing a fast decrease of the resistance in presence of the external stimulus [87]. The ${\mathrm{NO}}_{2}$-sensing properties of ${\mathrm{MAPbI}}_{3}$ thin films have been investigated both at ambient and high pressure (with argon from 1 to 8 MPa). Interestingly, the sensor presents a very low detection limit with a response even under extremely low ${\mathrm{NO}}_{2}$ concentrations (about 1 ppm) (Figure 17d) and an average sensitivity as high as 0.62 ppm${}^{-1}$ (inset of Figure 17d). In addition, the ${\mathrm{MAPbI}}_{3}$-based ${\mathrm{NO}}_{2}$ sensor exhibits a very quick-responsive character with average response and recovery times of about 5 s and 25 s, respectively (Figure 17e), good selectivity versus other gases, such as ${\mathrm{SO}}_{2}$, HCHO, ${\mathrm{CH}}_{4}$, CO, ${\mathrm{NH}}_{3}$, ${\left({\mathrm{CH}}_{3}\right)}_{3}N$, $O_{2}$ and $H_{2}O$, and interesting reproducibility, as the sensor response remains stable still after more than 12 cycles.

In order to enhance ${\mathrm{MAPbI}}_{3}$ gas sensing performances toward ${\mathrm{NO}}_{2}$ and acetone vapors, a doping process has been used, introducing thiocyanate ions (SCN-) into the perovskite lattice and obtaining ${\mathrm{CH}}_{3}{\mathrm{NH}}_{3}{\mathrm{PbI}}_{3-x}{\left(\mathrm{SCN}\right)}_{x}$ with x in the range 0.016–0.053 [49]. As in other reports, also Zhuang’s research points out how the film morphology results fundamental in the sensing process. A film thickness of 120 nm maximizes the electrical response compared to 170 and 210 nm thick layers (Figure 17a). Thinner layers indeed have an higher roughness and provide a larger surface to volume ratio, inducing a stronger sensing response. On the other hand, it is demonstrated how layers thinner than 120 nm do not uniformly cover the substrate, creating non conductive channels.

The detection limits of the 120 nm-film are found to be 20 ppm and 200 ppb for acetone and ${\mathrm{NO}}_{2}$, respectively, whereas its sensitivity is $5.6\cdot 10^{-3}\;{\mathrm{ppm}}^{-1}$ for acetone and $5.3\cdot 10^{-1}\;{\mathrm{ppm}}^{-1}$ for ${\mathrm{NO}}_{2}$ (Figure 17b,c). Recovery times also show differences between the two active means, obtaining 4 min for acetone and 1.5 min for ${\mathrm{NO}}_{2}$. Differences in detection limit and recovery times arise from a stronger (for acetone) or weaker (for ${\mathrm{NO}}_{2}$) interaction with the perovskite surface. However, it is interesting to notice how perovskite positively responds to the presence of both an electron-withdrawing (${\mathrm{NO}}_{2}$) and an electron-donating (acetone) gas, resulting in both cases in a conductivity enhancement. It is related to the ambipolar charge transport nature of hybrid metal halide perovskite, due to the similar effective masses of electrons and holes [97]. This sensor also exhibits excellent reproducibility and greatly improved environmental stability thanks to the chemical bound between SCN- ions and Pb atoms in perovskite lattice, compared to the non doped ${\mathrm{MAPbI}}_{3}$ perovskite.

The performance of ${\mathrm{MAPbI}}_{3}$ films as sensors has been tested with ethanol and other polar and non-polar organic species at room temperature [7]. The resistance of the film substantially decreases when it is exposed to VOC vapor and recovers back to high resistance when the VOC gas is removed and the surface is exposed to an inert gas. Ethanol exposure does not damage the ${\mathrm{MAPbI}}_{3}$ structure and the interaction between the perovskite and the gas is assumed to be mainly due to physisorption and limited on the perovskite grain boundaries. From the dynamic response–time plot of ${\mathrm{MAPbI}}_{3}$ film (Figure 17f), when it is alternately exposed to ethanol and inert gas, a response time of 66 s and a recovery time of 67 s have been inferred, which are faster than other type gas sensors [101]. In addition, the films are characterized by a sensitivity of $3\cdot 10^{-4}\;{\mathrm{ppm}}^{-1}$ and a limit of detection (LOD) of 1300 ppm, lower than 3300 ppm, immediately dangerous for life or health.

Moreover, tests with polar and non-polar organic VOCs indicate that generally the perovskite sensors are more sensitive to the former, resulting in higher responses. This behavior could be ascribed to the fact that the polar gas molecules attach stronger to ${\mathrm{MAPbI}}_{3}$ defect sites, since ${\mathrm{MAPbI}}_{3}$ perovskite itself is a polar ionic crystal.

Hybrid lead halide ${\mathrm{MAPbI}}_{3-x}{\mathrm{Cl}}_{x}$ films have been successfully tested as portable, flexible, self-powered, and ultrasensitive ozone sensors operating at room temperature [98].

Ozone ($O_{3}$) is considered one of the principal pollutants harmful to the human respiratory system, causing inflammation and congestion of the respiratory tract. Therefore it is necessary to control the ozone concentration in the atmosphere and in confined environments through the continuous monitoring of the gas. For this reason, the development of both effective and inexpensive methods and devices are required.

**Figure 18:**
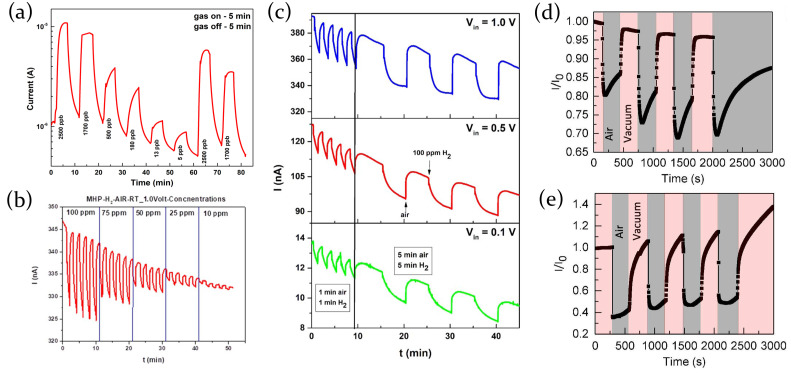
(**a**) Electrical response of ${\mathrm{MAPbI}}_{3-x}{\mathrm{Cl}}_{x}$ films under various ozone concentrations (5–2500 ppb). Adapted with permission from [98]. Copyright © 2017, American Chemical Society. https://pubs.acs.org/doi/10.1021/acssensors.7b00761. Further permission should be directed to the ACS. (**b**) Reversible current modulation for 300 nm thick mixed ${\mathrm{MAPbI}}_{3-x}{\mathrm{Cl}}_{x}$ perovskite films under exposure (for five minutes) in various hydrogen concentrations, revealing a detection limit of 10 ppm of $H_{2}$. (**c**) Current modulation of mixed ${\mathrm{MAPbI}}_{3-x}{\mathrm{Cl}}_{x}$ perovskite layers under $H_{2}$ and air flow, for various exposure times (one and five minutes) and under different forward biases (0.1, 0.5 and 1 Volt). Adapted with permission from [8] under the terms of the Creative Commons Attribution 3.0 license. Copyright © 2020, IOP Science. (**d**) Dark current and (**e**) photocurrent modulation for ${\mathrm{MAPbBr}}_{3}$ perovskite measured under vacuum and standard atmosphere. Reprinted from [71], with permission of AIP Publishing; Copyright © 2017.

The electrical resistance of the film promptly decreases when exposed to ozone with a response time between 188 and 225 s, depending on the $O_{3}$ concentration (with response time getting lower values as the concentration of the $O_{3}$ increases), and recovers to the pristine values within 40–60 s, after the complete removal of gas. In addition, tests have shown that the films are able to detect, with good repeatability, even ultralow ozone concentrations ranging from 2500 to 5 ppb, characterized by a reduction of the response magnitude with ozone concentration decrease (Figure 18a).

Exploiting the reversible interaction of mixed ${\mathrm{MAPbI}}_{3-x}{\mathrm{Cl}}_{x}$ perovskite with $H_{2}$ molecules, inducing a decrease of current in the film, Gagaoudakis et al. [8] have realized portable, flexible, self-powered, fast and sensitive hydrogen sensing elements, operating at room temperature. Concentrations of $H_{2}$ gas down to 10 ppm could be detected with a sensitivity depending on the concentration and included in the range from 0.3% at 10 ppm to 5.2% at 100 ppm (Figure 18b,c). Moreover, the sensing element film shows a good reversibility and quick response and recovery times with average values of 45 and 35 s, respectively.

As regarding the ${\mathrm{MAPbBr}}_{3}$ sensing properties, its interaction with air ($O_{2}$ and $H_{2}O$ molecules) has been found to induce a decrease of both photocurrent and dark current (Figure 18d,e), compared to vacuum atmosphere, accompanied in this case by a PL enhancement. Air infiltration in perovskite lattice is thought to be responsible for the formation of shallow trap states. Photo-generated carriers can then be trapped and form radiative recombination centers, inducing the decrease of both dark and photo current; on the other hand, charge carrier trapping is seen to reversibly enhance the sample PL emission [71].

Due to their superior stability at ambient conditions [102,103], totally inorganic perovskites have been explored as self-powered oxygen and VOCs sensors [104]. Porous ${\mathrm{CsPbBr}}_{3}$ layers with an average thickness of about 350 nm, constituted of interconnected grains of 100–200 nm in diameter, have been tested. It has been demonstrated that at room temperature and under visible light illumination, the photocurrent generated by these devices is highly sensitive to both oxidizing and reducing gases with very quick response and recovery time.

**Figure 19:**
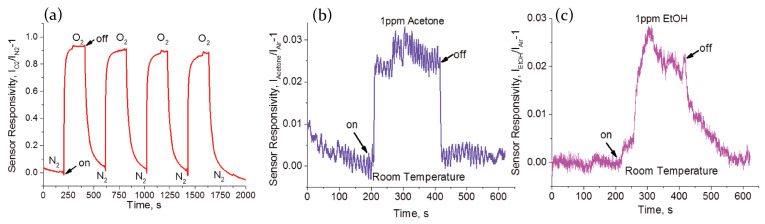
Dynamic ${\mathrm{CsPbBr}}_{3}$ sensor responsivity to (**a**) $O_{2}$, (**b**) acetone and (**c**) ethanol exposure at room temperature under visible-light irradiation. Adapted with permission from [104]. Copyright © 2017, WILEY-VCH Verlag GmbH and Co. KGaA, Weinheim.

The dynamic responsivity of the ${\mathrm{CsPbBr}}_{3}$ devices when exposed to $O_{2}$, acetone and ethanol (Figure 19a–c) indicates that the photocurrent rapidly increases and stabilizes upon gas injection, and quickly recovers its initial value after switching back to the pure $N_{2}$ atmosphere. Response time and recovery time of 17 and 128 s, respectively, have been estimated for oxygen, while 9.8 s and 5.8 s for acetone. Moreover the ${\mathrm{CsPbBr}}_{3}$ devices could easily detect down to 1 per cent, and possibly lower content, of $O_{2}$ in $N_{2}$ and concentrations as low as 1 ppm of acetone and ethanol. Finally, the sensor reveals a good repeatability when exposed to consecutive cycles of gas exposition, and after two weeks storage, confirming that they are compelling alternatives to oxide-based sensors.

In the Table 1, the main sensing properties of the described perovskite devices are reported with some information about the active material (chemical composition and structure) and the target gas which induces the electrical response.

$T_{\mathrm{res}}$ and $T_{\mathrm{rec}}$ refer to the response and recovery time, defined as the time needed to reach the 90% of the final value and the time to fall to 10% of the final value after removing the gas source, respectively. The last column shows instead the limit of detection (LOD), i.e., the minimum gas quantity the sensor is able to detect.

Concerning the sensitivity (*S*), in literature there is not a common definition, so some disorder can be generated and a clarification is needed. Some authors refer to *S* as the ratio $\frac{\Delta I}{I_{0}}=\frac{I-I_{0}}{I_{0}}$ where *I* represents the current value obtained in presence of the target analyte and $I_{0}$ is the reference value in inert atmosphere [8,66,104]. Similarly, *S* can be considered as the ratio $\frac{\Delta R}{R_{0}}=\frac{R-R_{0}}{R_{0}}$, where the resistance change is evaluated [81]. So they provide *S* value with a reference gas concentration. Others distinguish between $R_{S}$ (the sensor response) and *S*, specifying that the first one refers to the above mentioned ratio $\Delta I/I_{0}$[7,49] or the resistance ratio $R/R_{0}$[87], whereas the *S* represents the sensor response ($R_{S}$) divided by gas concentration, expressed in ${\mathrm{ppm}}^{-1}$. In these cases, sensitivity values are not dimentionless and often refer to average values [49,87]. A last sensitivity definition comes from [98] where *S* is defined as the ratio $I_{max}/I_{min}$ where the terms represents the maximum and the minimum values the current reaches in the on/off gas cycle. Finally, some authors do not report any sensitivity value [71,84].

### 3.2. Optical Sensors

Even if the metal halide perovskite resistive response to the presence of a particular gas is the most studied thus far, recently some reports have highlighted how the presence or absence of a particular analyte can induce the activation or deactivation of some relaxation processes, and consequently, determine a change in the sample photoluminescence emission. Having a deeper insight into the issue can represent the starting point for the application of lead halide perovskite as luminescent-based gas sensor. The interaction between perovskite films and gases of different nature has been proved to induce modification both of the visual aspect of the film, noticeable as a color change, and a PL modulation, which returns to the initial value once the gas source is removed.

#### 3.2.1. Color Change Gas Sensor

The first report of a reversible color change from perovskite films interacting with the surrounding atmosphere dates back to 2013 with Zhao’s experimental analysis [85]. The exposition of ${\mathrm{MAPbI}}_{3}$ perovskite films to ammonia gas was found to lead to a rapid (<1 s) change in its color from brown to transparent, and then turn back to its pristine condition upon removing the ${\mathrm{NH}}_{3}$ gas. XRD measurements highlighted structural changes in perovskite crystals, likely due to the formation of an intercalation compound or a coordination complex from perovskite and ${\mathrm{NH}}_{3}$ interaction. Since similar results have been found also for ${\mathrm{MAPbI}}_{3-x}{\mathrm{Cl}}_{x}$ and ${\mathrm{MAPbBr}}_{3}$ perovskites, the discovery opened a route for a potential use of lead halide perovskite as optical sensor application.

**Figure 20:**
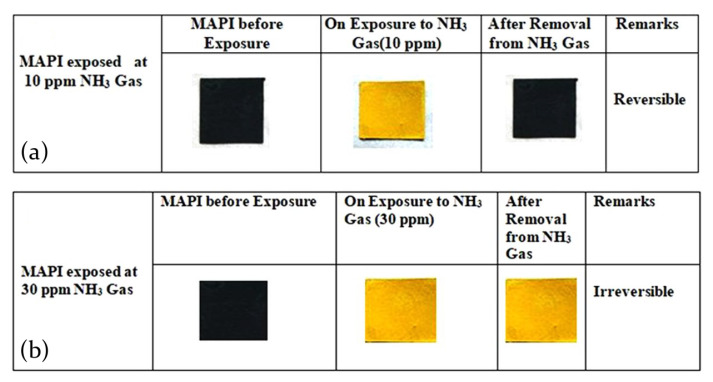
Photographs of the ${\mathrm{MAPbI}}_{3}$ film exposed to (**a**) 10 ppm ${\mathrm{NH}}_{3}$ gas, which allows a reversible process, and to (**b**) 30 ppm, which induces an irreversible film transformation. Adapted with permission from [80] under the terms of the Creative Commons Attribution 4.0 International License. Copyright © 2018, Springer Nature.

Later, Maity’s group developed a paper-based gas sensor in which ${\mathrm{MAPbI}}_{3}$ perovskite film changes its color from pristine black to yellow when exposed to ${\mathrm{NH}}_{3}$ vapors (Figure 20a) [80]. Contrary to what previously stated, color change has been ascribed to ${\mathrm{PbI}}_{2}$ molecules formation, confirmed by XRD measurements.

Unfortunately, both studies highlighted the presence of an exposition limit in terms of time and gas concentration, above which the color change process becomes irreversible due to the perovskite film degradation (Figure 20b).

#### 3.2.2. PL Emission-Based Gas Sensors

The atmosphere around a perovskite sample has been proved to be crucial for the modulation of the photoluminescence emission. Oxygen, water molecules or other volatile compounds are indeed verified to be responsible, for example, for trap states passivation processes, leading to an increase of the photoluminescence intensity, or modification of the chemical structure of the perovskite, inducing a worsening of the optical properties of the film.

In case of controlled atmosphere inside a test chamber, the effect of a single source can be evaluated, as it happened for the experimental analysis of Howard et al. [69], who demonstrated how humidity alone can induce perovskite degradation through the formation of hydrated species or facilitate ion migration. Besides, the combined effect of two analytes has also been evaluated.

The role of oxygen and water molecules on the photoluminescence properties of hybrid ${\mathrm{MAPbI}}_{3}$ perovskite films has been subject matter of the research for Galisteo-Lopez et al. [65], evidencing that the PL activation is related to the passivation of trap states by $O_{2}$ molecules, whereas a humid atmosphere is responsible for the photodarkening process. This feature is evident considering the PL maximum at 775 nm as a function of illumination time, for a sample exposed to air under continuous optical 500 nm excitation. As shown in Figure 5a, the PL peak shows an initial increase, reaches its maximum value at $t_{c}$ and then decreases for higher illumination times. Moreover, both photoactivation and photodarkening processes are demonstrated to be dependent on the pump intensity; for increasing excitation intensities, indeed, the rate of photoactivation process increases and $t_{c}$ becomes smaller, reaching a stationary behavior after 0.7 $W/{\mathrm{cm}}^{2}$ pump intensity. However, the emission spectral shape has been evaluated at different excitation times, for a fixed pump intensity, evidencing that it remains unchanged. This feature, together with the fact that no color change of the film has been observed, allows one to exclude any permanent degradation process of the film.

A possible explanation of the observed experimental results involves the presence of ${\mathrm{Pb}}^{2+}$ ions on the pristine perovskite surface, which act as trap sites for photoexcited charges, reducing the number of charges available for radiative recombination processes. Once oxygen molecules are injected in the test chamber, they passivate such trap states with the formation of lead oxide, leading to the PL intensity increase. On the other hand, moisture in atmosphere induces the formation of hydrated species, which weaken the hydrogen bonds between organic cations and ${\mathrm{PbI}}_{6}$ octahedra and support the formation of complexes with water molecules. Since it does not completely destroy the perovskite crystal structure, a recovery of the photoluminescence is possible switching the atmosphere from air to pure oxygen under illumination (Figure 5b).

Air molecules have been demonstrated to act as passivating agents also for bromide based hybrid lead halide perovskites, affecting the photoluminescence emission [70,71]. The result is a PL enhancement when the external atmosphere is switched from vacuum to air. However, differently from the above mentioned research [65], $O_{2}$ and water contributions are not separated.

In particular, Fang et al. [70] observed a drastic photoluminescence decrease passing from air to vacuum atmosphere, the emission going down to 10% of the initial maximum value after 120 s. Since the effect of the exposure to the gas can be reset by evacuating the test chamber, it implies the interaction is governed by physical rather than chemical processes. With the aim of checking the selectivity of the response, the ${\mathrm{MAPbBr}}_{3}$ single crystal samples have been exposed to other gas sources (dry $N_{2}$, ${\mathrm{CO}}_{2}$ and Ar); however it was found that they do not produce any effects on the PL intensity. Afterwards, the authors investigated the trap healing mechanism in order to have a deeper knowledge about the position of the traps inside the crystal.

**Figure 21:**
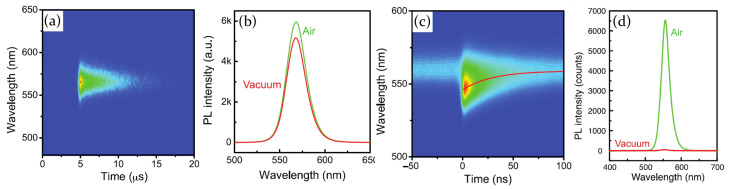
(**a**) 2D pseudocolor plot of TRPL under 800 nm femtosencond laser excitation in air. (**b**) Two-photon (800 nm) excited PL spectra measured in air and in a vacuum. (**c**) 2D pseudocolor plot of TRPL under 400 nm excitation; the red line indicates the emission peak wavelength. (**d**) PL spectra registered in a vacuum and in air, under 400 nm laser excitation. Adapted with permission from [70] under the terms of the Creative Commons Attribution-NonCommercial license. Copyright © 2016, American Association for the Advancement of Science.

In this regard, both 400 nm- and 800 nm-laser excitations have been used, the latter allowing to obtain a two-photon excitation and interaction with the innermost regions of the crystal below the surface. Unlike in the 400-nm excitation experiment (Figure 21d), the PL intensity modulation under atmosphere change has been found to be much smaller under two-photon excitation (Figure 21b), concluding that the photocarrier recombination is strongly affected by the surface properties (Figure 21a–d). Additional evidence comes from 2D pseudoplots of the time resolved photoluminescence (TRPL) under 800 nm and 400 nm excitation, as shown in Figure 21a,c. The latter indeed evidences that the emission peak wavelength suffers a redshift with ongoing illumination time, whereas in the 800 nm excitation case the emission wavelength remain unaltered, proving that the traps are located near the surface of the film rather than in the bulk.

Moreover, the PL quenching observed from the exposition of ${\mathrm{MAPbBr}}_{3}$ single crystals from air to vacuum has been noticed to have a corresponding current modulation, revealing a positive correlation between the resistance of the sample and the air pressure [71]. As a consequence, both dark current and photocurrent have been shown to increase in vacuum. This behavior relates to the fact that air infiltration in perovskite lattice can form shallow trap states close to the perovskite band edges. Those states can trap photo generated carriers, acting as radiative recombination centers. Therefore, in presence of air the PL intensity enhances and a smaller quantity of carriers is available for the conduction, resulting in a current decrease.

The interaction with ammonia, on the contrary, induces structural changes in hybrid lead halide perovskite, regardless of the chemical composition. Ruan et al. [83], for example, realized ${\mathrm{MAPbX}}_{3}$ (X = I, Br, Cl) single crystals and washed them with a 30 wt.% ammonia solution, revealing an instantaneous color change for all the samples, which indicates that a new phase on the film is formed. Despite XRD measurements indicated that ${\mathrm{MAPbX}}_{3}$ structure is recovered after an annealing process post-treatment, it evidences how the halogen size influences the stability of the material. Cl presence in ${\mathrm{MAPbCl}}_{3}$ single crystals, indeed, not only leads to a contracted crystal unity cell, but also determines a more stable perovskite structure compared with the larger unit cell of ${\mathrm{MAPbI}}_{3}$, which shows a complete phase change after ammonia treatment.

Among the three samples, ${\mathrm{MAPbBr}}_{3}$ showed an almost complete restoration of the XRD peaks after the ammonia treatment; as a consequence, it has been chosen as active material for the realization of the ${\mathrm{NH}}_{3}$ sensor. In particular, its photoluminescence decreases of 60% in 2 s when exposed at low ammonia concentration (vapor from 0.3 wt.% NH${}_{3}$ solution), and recovers the initial level in 20 s after removing the ${\mathrm{NH}}_{3}$ source. However, for higher gas concentrations the sensing response has been found to be slower, reaching 45 s and 1300 s as recovery time for 1 wt.% and 3 wt.% NH${}_{3}$ solution, respectively. The sensor has then been tested with other volatile compounds, such as water, methanol, ethanol and acetone and the response has been measured as the ratio $\Delta I/I_{0}$, where ΔI represents the variation of PL intensity with and without the external stimulus. Methanol, ethanol and acetone interaction resulted in a PL increase of 25%, 5% and 8%, respectively, whereas water vapor determined a 32% luminescence quenching.

Hybrid lead halide perovskites have also been tested for the detection of volatile organic compounds, exploring the role of the chemical nature of the target gas and its influence on the response of the active mean.

**Figure 22:**
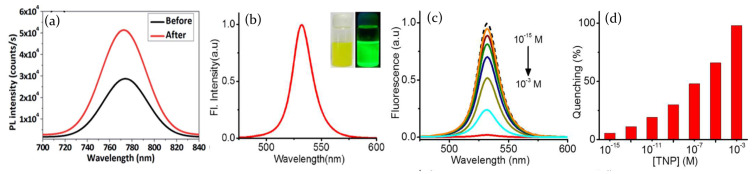
(**a**) Photoluminescence intensity of ${\mathrm{MAPbI}}_{3}$ perovskite film before and after ethanol gas exposure. Adapted with permission from [7] under a Creative Commons Attribution-NonCommercial 3.0 Unported license. Copyright © 2020, Royal Society of Chemistry. (**b**) Emission spectrum of ${\mathrm{MAPbBr}}_{3}$ nanoparticles. Inset: photographs of the nanoparticle suspension in toluene under room light (left, yellow colored) and 364 nm UV light (right, green colored). (**c**) Fluorescence spectra of ${\mathrm{MAPbBr}}_{3}$ nanoparticles at an increasing concentration of TNP, ranging from $10^{-3}$ M to $10^{-15}$ M, with the (**d**) corresponding quenching percentage for the increasing TNP concentration. Used with the permission of the Royal Society of Chemistry, from [105]. Copyright © 2014. Permission conveyed through Copyright Clearance Center, Inc.

The sensing mechanism of the ${\mathrm{MAPbI}}_{3}$ perovskite devices proposed by Nur’aini et al. [7] for VOCs is supported by the photoluminescence (PL) measurements (Figure 22a). Adsorption of VOC gas molecules to vacancies in ${\mathrm{MAPbI}}_{3}$ film leads to trap state passivation. Before ethanol exposure, high density of crystal defects is indicated by low PL intensity, whereas during the exposition the defect density becomes lower and crystallinity of perovskite improves, leading to the PL intensity increases.

From the study of Kim et al. [96], focused on the interaction between ${\mathrm{MAPbBr}}_{3}$ and aliphatic amine gases (${\mathrm{EtNH}}_{2}$, ${\mathrm{Et}}_{2}\mathrm{NH}$ and ${\mathrm{Et}}_{3}N$) (Figure 13c), effective naked-eye fluorescence sensors based on ${\mathrm{MAPbBr}}_{3}$ nanoparticle films have been realized. The exposition to all amines has been observed to lead to PL quenching with changes in intensity of strong green fluorescence; nevertheless the process is fully reversible only for ${\mathrm{Et}}_{3}N$ exposure with very fast recovery time (<1 s).

${\mathrm{MAPbBr}}_{3}$ nanoparticles are used as excellent optical sensors for the detection of picric acid (2,4,6-trinitrophenol, TNP), potent explosive for lethal weapons, with high selectivity and sensitivity both in solution and vapor state [105]. The nanoparticles in toluene, yellow-colored under room light, emit bright green fluorescence when irradiated with UV light at 364 nm (inset of Figure 22b) and the solution shows quenching of the fluorescence (up to 97%) almost instantaneously when TNP is added (Figure 22c,d). Moreover the perovskite nanoparticles are very sensitive and are able to detect TNP with a very low limit of detection, down to femtomolar (10${}^{-15}$ M) concentrations. The response is due to the properties of TNP that, being a good electron acceptor, quenches the perovskite fluorescence efficiently. A key role on the overall detection mechanism is played by the hydroxyl group which interacts with the perovskite nanoparticles through the formation of very stable hydrogen bonding.

Recently, also fully inorganic perovskites have been explored as optical sensors because they exhibit higher long term stability with respect to the hybrid perovskites.

**Figure 23:**
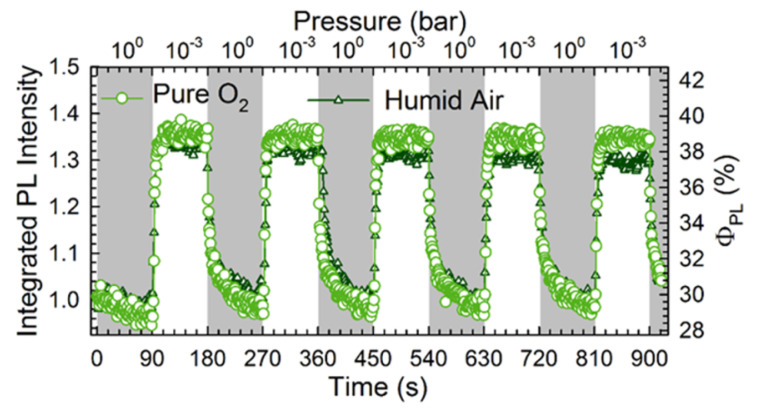
Integrated PL intensity and corresponding PL quantum yield ($\Phi_{\mathrm{PL}}$) of ${\mathrm{CsPbBr}}_{3}$ NCs for $O_{2}$/vacuum and humid air/vacuum cycles between P = 1 bar (gray shades) and P = 10${}^{-3}$ bar (white). Adapted with permission from [79]. Copyright © 2017, American Chemical Society. https://pubs.acs.org/doi/10.1021/acs.nanolett.7b01253. Further permission should be directed to the ACS.

Based on the results inferred from the investigations on the interaction between fully inorganic perovskites and ambient air, ${\mathrm{CsPbBr}}_{3}$ perovskite nanocrystals have been tested as oxygen sensing materials [79] and PL measurements have revealed a strong emission quenching. Actually, the photoluminescence emission modulation in presence of $O_{2}$ rich atmosphere is due to the direct extraction of photogenerated electrons from the conduction band of the NCs causing the PL quenching (Figure 23).

Differently, the photoluminescence modulation in ${\mathrm{CsPbBr}}_{3}$ single crystals in presence of oxygen [106,107] is an effect of the trap state passivation and through theoretical calculations the nature of these trap states has been investigated. The presence of bromide vacancies on the perovskite surface determines the formation of shallow energy levels at the bottom of the conduction band minimum (CBM) that could trap charge carriers and give rise to non-radiative recombination. When $O_{2}$ molecules are adsorbed at the location of bromide vacancies, a charge redistribution happens and the shallow trap states at the bottom of the CBM are removed, inducing an increase in the photoluminescence emission.

Recent researches report fully inorganic perovskites as sensing material for the detection of ammonia and explosive vapors, confirming the idea that these materials could represent the ideal candidates for the realization of optical sensors.

**Figure 24:**
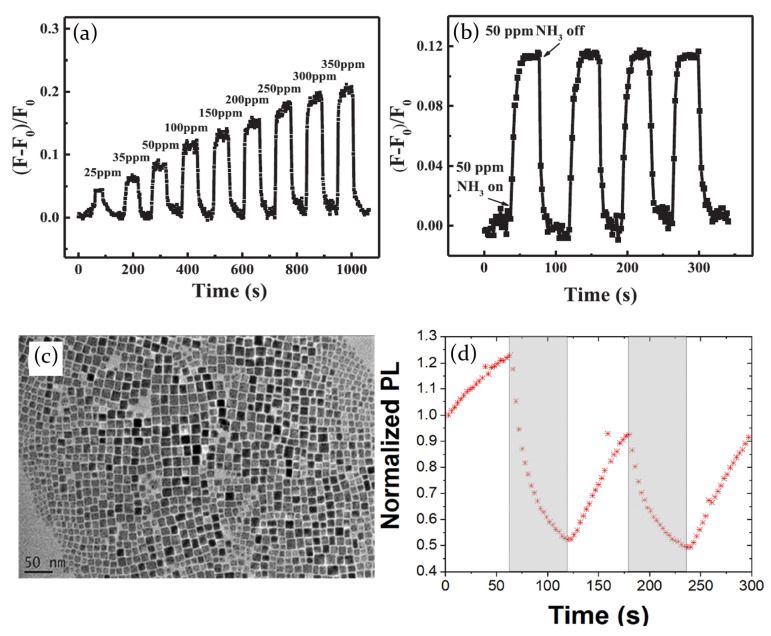
(**a**) Dynamic sensing response of ${\mathrm{CsPbBr}}_{3}$ quantum dots (QDs) for different ${\mathrm{NH}}_{3}$ concentrations (from 25 to 350 ppm). (**b**) Four successive sensing cycles of the ${\mathrm{CsPbBr}}_{3}$ QDs at 50 ppm. Adapted with permission from [86]. Copyright © 2020, WILEY-VCH Verlag GmbH and Co. KGaA, Weinheim. (**c**) Transmission electron microscopy images of ${\mathrm{CsPbBr}}_{3}$ nanocrystals. (**d**) Photoluminescence emission as a function of time for a ${\mathrm{CsPbBr}}_{3}$ nanocrystal film under repeated cycles of exposure to pure nitrogen (white areas) and DNT vapors in $N_{2}$ (gray areas). Adapted with permission from [108] under a Creative Commons Attribution (CC BY) license. Copyright © 2020, AIP Publishing.

Contrary to results obtained with hybrid perovskites, ${\mathrm{CsPbBr}}_{3}$ based sensor works in a PL turn-on mode rather than in a PL quenching one [86]. The exposition of the sample to ammonia gas reveals an incredible photoluminescence intensity increase of the QDs (about three times than the original one) and the pristine condition is restored when the gas source is removed (Figure 24a). Moreover, TEM and XRD measurements reveal that the size and morphology of the QDs remain the same after treatment, and the crystalline structure is retained after being exposed to ammonia gas. It suggests the interaction between perovskite and ${\mathrm{NH}}_{3}$ is physical rather than chemical, where ammonia molecules passivate surface defects of perovskite QDs, reducing the non radiative component from 18.78% to 12.73%. The sensor is shown to have a low detection limit of 8.85 ppm and a response and recovery times of about 10 s and 30 s, respectively, for a 50 ppm ammonia concentration (Figure 24b). The selectivity of the active material has also been tested, first evaluating the effect of $O_{2}$ molecules, then a variety of other gases (such as acetone, water, isopropanol, HCl, ethanol, ${\mathrm{CO}}_{2}$), all of them inducing just a slightly decrease of the photoluminescence intensity.

The effect of the dimensionality of the sensing material has instead been analyzed by Harwell et al. [108], evidencing how by tuning the perovskite dimensionality from 3D (bulk ${\mathrm{CsPbBr}}_{3}$) to 2D (layered phenethylammonium ${\left(\mathrm{PEA}\right)}_{2}{\mathrm{Cs}}_{2}{\mathrm{Pb}}_{3}{\mathrm{Br}}_{10}$) and finally to 0D (${\mathrm{CsPbBr}}_{3}$ nanocrystals) structures can dramatically enhance the response of the films to external stimuli. ${\mathrm{CsPbBr}}_{3}$ nanocrystals (Figure 24c), thanks to their low dimensionality, have a high surface to volume ratio, which makes them efficient for the detection of 2,4-dinitrotoluene (DNT) vapors through photoluminescence quenching. Contrary to what observed for 3D and 2D perovskite structures, CPB nanocrystals have been indeed seen to respond to the presence of DNT vapors with a rapid drop in PL in about 1 min, and recover about 50% of the emission when returning to pure nitrogen atmosphere (Figure 24d). The reversibility of the PL response to the exposure to DNT vapors is probably due to the presence of ligands on the perovskite NC surface, which prevent DNT molecules from strongly binding to the perovskite. As a consequence, when the gas source is removes, the analyte molecules can easily desorb and the pristine condition is restored.

Contrary to what we have seen for perovskite resistive sensors, the study on photoluminescence modulation based sensors, both with hybrid and fully inorganic perovskites, is not furthered yet. Physical and chemical mechanisms behind the interaction between perovskite and different analytes are not clear so far. Literature lacks of a systematic and extensive study of sensing properties, allowing to identify a good sensing material thanks to parameters such as response and recovery times, sensitivity and limit of detection. Up to now, most authors have provided qualitative researches, showing the potential response of the active mean to a determined analyte in terms of increase or quenching of the photoluminescence. However, it could represent a starting point for a future research involving a complete study of the sensing properties of both hybrid and promising fully inorganic perovskites, with the aim to have a better insight into the question and develop an efficient gas sensor.

## 4. Perspectives

As we have extensively discussed, the interaction of the perovskites with the ambient, when does not lead to reversible effects, is detrimental to their sensing properties. So improving the performance of gas sensing devices requires specific expedients to reduce their degradation over time and when subjected to external stimuli.

To this aim, perovskites with different chemical compositions, dimensionality and surface morphology, or passivation strategies could be employed for the optimization and the stabilization of the sensing devices.

Chemical action on the perovskite composition can improve the sensing ability of the active material—for example, through a doping process [49] or by simply modifying the halogen, which has been demonstrated to influence both the stability and the response speed [83].

Moreover, the deposition process can change the morphology properties of the resulting film (such as the thickness and the substrate coverage) [49,66] affecting, in turn, the device sensitivity. Additionally, the dimensionality of the perovskite can strongly influence the response of the device and it has been shown that switching from 3D to 0D structures allows one to obtain an higher sensitivity to the external stimuli thanks to the 0D greater surface to volume ratio [108].

Furthermore, post-deposition processes, such as annealing treatments, have been seen to improve the stability of the perovskite against moisture, and consequently, enhance the sensitivity of the perovskite toward environmental gases [70].

Operational stability improvement of lead halide perosvkites has been proved also for drop cast CsPbBr${}_{3}$ NC thin films deposited on hexamethyldisilazane (HMDS) hydrophobic functionalized substrates. These results are ascribed to a closer NC packing in the films on treated substrate, allowing the reduction of the film interaction with external moisture [16].

In the research of new sensing systems, with the aim of the device sensitivity enhancement, another route is open.

In PL-based optical sensors, the photoluminescence of the active mean is sensitive to the surrounding gas partial pressure with quenching or enhancement of emission in presence of a target gas. It is proven that in some materials (mostly polymers) exhibiting amplified spontaneous emission (ASE), the sensing sensitivity can be further boosted by harnessing the ASE and the lasing action.

Therefore, novel methods of gas sensing based on ASE or lasing in perovskites should be explored and proposed.

It has been demonstrated that metal-lead halide perovskites exhibit good light amplification emission. Evidence of high optical gains and efficient amplified spontaneous emissions (ASE) is reported for bulk polycrystalline thin films of organic- inorganic [29,31,109,110] and fully inorganic perovskites [33,35] and for perovskite nanocrystal films [16,38,111,112,113,114].

However, it has been observed that laser irradiation in vacuum of some perovskites thin films could result in ASE degradation [30]. The correlation between the observed ASE operational stability and the irradiation-induced variations in the local morphology and emission properties, observed at high excitation energy density, suggests that the main process leading to ASE quenching is related to the film melting induced by localized heating. Instead, the photodegradation lessens at lower excitation energy density. Surprisingly, ASE properties, such as intensity and stability, are improved when the film is irradiates in air, indicating that the interaction with oxygen has overall positive effects on the emission properties.

In spite of that, the influence of ambient gas on the perovskite active material that leads to the ASE intensity modulation is to date almost unexplored. Otherwise, the gas-induced modulation of ASE intensity has been already observed and studied in other materials.

ASE sensitivity to an external stimulus was demonstrated since 2005 [115] in semiconducting organic polymer. Trace vapors of the explosives 2,4,6-trinitrotoluene (TNT) and 2,4-dinitrotoluene (DNT) induce ASE attenuation during irradiation of thin films due to the introduction of non-radiative deactivation pathways competing with stimulated emission. Attenuation of ASE intensity is observed when the organic polymer thin films are irradiated in presence of TNT vapors down to 5 ppb, showing a sensitivity to the explosives, which is higher when films are pumped at energy densities near the lasing threshold, up to more than 30 times higher than that observed from spontaneous emission.

Analogously, also oligofluorene based compounds, such as Ter(9,9-diarylfluorene) (TDF) films, are demonstrated to be sensitive to the surrounding oxygen partial pressure with fast response, reversibility and high efficiency [116]. The PL emission intensity is quenched by the presence of O${}_{2}$, while the emission spectral features, such as peak wavelength, vibronic progression and bandwidth, are unaffected. Beside it has been shown that sensitivity of a chemical sensor can be enhanced in both amplified spontaneous emission and lasing action. In particular, ASE attenuation is found to be 10 times higher than spontaneous emission quenching and enhanced up to 20-fold in lasing action.

The experimental evidence of the enhanced sensitivity of an optical gas sensor when the ASE or the lasing action are monitored, instead of the PL emission, stimulates the exploration of the response of perovskites at excitation densities overcoming the ASE threshold. Indeed, similar sensing improvements could be expected in perovskites optical sensors, but such results are still missing in recent literature.

To obtain high-performance sensing devices it is mandatory to have active materials with high operation stability. For this reason, the stability improvement of light emission, amplification and lasing is an issue that is receiving growing attention. To enhance the ASE operational stability, the deposition of encapsulating layers, such a poly(methyl methacrylate) (PMMA) layer, followed by epoxy resin and glass encapsulation [109], or a commercial fluoropolymer layer (CYTOP) [36], has been proposed for MAPbBr${}_{3}$ bulk polycrystalline thin films. However, these expedients are not suitable for sensing devices, since the top layers prevent the interaction of the active material with the surrounding gases.

An alternative strategy is the treatment of the surface or, in some cases, of the substrate with functionalization chemicals.

As an example, concerning NC films an ASE stability improvement has been reported in films of MAPbBr${}_{3}$ nanocrystals treated with benzyl alcohol [113]. The addition of benzyl alcohol during the synthesis positively affects the structural and photophysical properties of the MAPbBr${}_{3}$ nanocrystals resulting in very stable nanocrystals and nanocrystal thin films, even after 4 months storage under ambient conditions, exhibiting near-unity PLQY, high optical gain (520 cm${}^{-1}$) and ultralow ASE thresholds (∼13.9 μ J cm${}^{-2}$) under femtosecond excitation.

It has been demonstrated also that a hydrophobic functionalization of the substrates with HMDS improves the ASE properties of drop cast ${\mathrm{CsPbBr}}_{3}$ nanocrystal thin films with a decrease of threshold down to 45%, an optical gain increase of up to 1.5 times and an ASE operational stability increase of up to 14 times [16].

In future, the employment of functionalized perovskites with good ASE properties, high stability and remarkable sensitivity to ambience, could lead to the realization of high-performance devices, by monitoring the ASE and the laser emission in the presence of target gases.

## Figures and Tables

**Table 1:** Sensing properties for perovskite-based resistive sensors described in this review. Data are grouped taking into account the perovskite active mean. The morphology of the film, the target gas used as analyte and the main sensing properties are reported.

Perovskite	Structure	Target Gas	$T_{\mathrm{res}}$	$T_{\mathrm{rec}}$	Sensitivity	LOD	Ref.	
${\mathrm{MAPbI}}_{3}$	Polycrystalline	$O_{2}$	0.4 s		3000		[66]	
${\mathrm{MAPbI}}_{3}$	Thin film	${\mathrm{NH}}_{3}$	3 s	4.5 s			[84]	
${\mathrm{MAPbI}}_{3}$	Nanorods	${\mathrm{NH}}_{3}$	≃130 s	≃120 s	≃90%	1 ppm	[81]	
${\mathrm{MAPbI}}_{3}$	Thin film	${\mathrm{NO}}_{2}$	5 s	25 s	0.62 ppm${}^{-1}$	1 ppm	[87]	
$\mathrm{SCN}-{\mathrm{MAPbI}}_{3}$	Thin film	${\mathrm{NO}}_{2}$	3.7 min	6 min	$5.3\cdot 10^{-3}$ ${\mathrm{ppm}}^{-1}$	200 ppb	[49]	
		Acetone	4.5 min	4 min	$5.6\cdot 10^{-3}\;{\mathrm{ppm}}^{-1}$	20 ppm	
${\mathrm{MAPbI}}_{3}$	Thin film	Ethanol	66 s	67 s	$3\cdot 10^{-4}\;{\mathrm{ppm}}^{-1}$	1300 ppm	[7]	
${\mathrm{MAPbI}}_{3-x}{\mathrm{Cl}}_{x}$	Thin film	$O_{3}$	188 s	40 s	9.69	5 ppb	[98]	
${\mathrm{MAPbI}}_{3-x}{\mathrm{Cl}}_{x}$	Thin film	$H_{2}$	39 s	61 s	0.3%	10 ppm	[8]	
${\mathrm{MAPbBr}}_{3}$	Single crystal	Air					[71]	
${\mathrm{CsPbBr}}_{3}$	Nanocrystals	$O_{2}$	17 s	128 s	0.93	10,000 ppm	[104]	
		Acetone	9.8 s	5.8 s	0.03	1 ppm	
		Ethanol			0.025	1 ppm	
